# Genomic characterization of invasive disease-causing Streptococcus pneumoniae in Lebanon, 2003–2025

**DOI:** 10.1099/mgen.0.001664

**Published:** 2026-03-13

**Authors:** Fatima Dakroub, Alannah C. King, Fata Akl, Alissar Zaghlout, Nancy Hourani, Harry Hung, Sarah Barada, Celina F. Boutros, Ana D. S. Ferreira, Lesley McGee, Lina Reslan, Stephanie Damaj, Nada Ghosn, Ghassan M. Matar, George F. Araj, Antoine Abou Fayad, Stephen D. Bentley, Stephanie W. Lo, Ghassan S. Dbaibo, for the Lebanese Inter-Hospital Pneumococcal Surveillance Program investigators, Nour Nahhouli

**Affiliations:** 1 Center for Infectious Diseases Research (CIDR) and WHO Collaborating Center for Reference and Research on Bacterial Pathogens, American University of Beirut, Beirut, Lebanon; 2 Parasites and Microbes Programme, Wellcome Sanger Institute, Hinxton, UK; 3 Department of Experimental Pathology, Immunology, and Microbiology, Faculty of Medicine, American University of Beirut, Beirut, Lebanon; 4 Institute of Microbiology and Infection, College of Medicine and Health, University of Birmingham, Edgbaston, Birmingham, B15 2TT, UK; 5 Division of Bacterial Diseases, Centers for Disease Control and Prevention (CDC), Atlanta, GA, USA; 6 Epidemiological Surveillance Unit, Lebanese Ministry of Public Health, Beirut, Lebanon; 7 Department of Pathology and Laboratory Medicine, American University of Beirut Medical Center, Beirut, Lebanon; 8 Milner Center for Evolution, Department of Life Sciences, University of Bath, Bath, UK; 9 The Great Ormond Street Institute of Child Health, University College London, London, UK; 10 Department of Pediatrics and Adolescent Medicine, American University of Beirut, Faculty of Medicine, Beirut, Lebanon

**Keywords:** antimicrobial resistance, genomics, invasive pneumococcal disease (IPD), pneumococcal conjugate vaccines (PCVs), pneumococcal lineage, *Streptococcus pneumoniae*

## Abstract

**Background*****.** Streptococcus pneumoniae* is a major human pathogen responsible for invasive pneumococcal diseases (IPDs). We utilized whole-genome sequencing to assess the impact of pneumococcal conjugate vaccines (PCVs) on the pneumococcal population causing IPD in Lebanon.

**Methods.** *S. pneumoniae* isolates collected between 2003 and 2025 (*n*=273) were sequenced and included in the study, which was divided into three periods. Private-PCV7 (2003–2009) and Private-PCV13 (2010–2015) correspond to the periods when the PCV7 and PCV13 vaccines were available only in the private healthcare sector, respectively. EPI-PCV13 (2016–2025) represents the period following PCV13 incorporation into the Expanded Program on Immunization (EPI). The Global Pneumococcal Sequencing (GPS) genome analysis pipeline was used to infer serotypes, genetic lineages, pilus locus and antimicrobial resistance (AMR) for 19 antibiotics. Phylogeny was constructed based on SNPs across the pneumococcal genome.

**Results.** A total of 58 GPS clusters (GPSCs) expressing 40 serotypes were identified. Overall, serotypes 3, 14 and 19F were the most prevalent serotypes, while GPSC6, GPSC12 and GPSC1 were the most predominant pneumococcal lineages. We detected a significant increase in non-vaccine types (NVTs) after PCV7 and PCV13 introduction. In contrast, PCV7 serotypes declined significantly over the three study periods. Collectively, PCV7 serotypes were associated with significantly higher mortality (31.1%) compared to NVT (15.9%). Moreover, IPD-associated mortality was significantly higher among older adult patients (33.3%) compared to children aged ≤5 years (12.1%). Non-susceptibility to penicillin was the most prevalent resistance (62.1%), and multidrug resistance (MDR; non-susceptibility to at least three antibiotics) was identified in 36.4% of the isolates. MDR was primarily driven by GPSC1, GPSC9, GPSC6 and GPSC10. A significant decline in MDR and AMR against seven antibiotics was observed in the EPI-PCV13 period compared to previous study periods.

**Conclusions.** Genomic surveillance is robust for tracking current NVT and identifying lineages that may influence future IPD trends in Lebanon. Given the high mortality rate detected in older adult IPD patients, implementing a routine immunization programme in this population may be beneficial.

**Impact Statement**

This is the first study from Lebanon to characterize invasive *S. pneumoniae* strains using whole-genome sequencing, analysing 273 isolates from 2003 to 2025. We demonstrate how vaccination policies have shaped circulating pneumococcal serotypes and lineages and antimicrobial resistance patterns over time. Our findings contribute to the understanding of the genetic structure of the *S. pneumoniae* population in Lebanon and inform future vaccination strategies for the control of invasive pneumococcal disease (IPD). We show that the recent replacement of PCV13 with PCV10 (Pneumosil) in the Expanded Program on Immunization may substantially reduce the vaccine coverage of circulating serotypes. Additionally, we underscore the potential benefits of targeted pneumococcal vaccination in older adults, who exhibited the highest IPD-related mortality in the study. Despite ongoing economic and regional instabilities, our surveillance efforts have remained active and continue to capture changes in the pneumococcal population during and after PCV rollout.

## Data Availability

Genome sequences analysed in this study have been deposited in the European Nucleotide Archive under study accessions ERP001505 and PRJEB88880. The accession numbers for individual genomes were submitted as supplementary files. An interactive visualization of the phylogeny and metadata associated with the pneumococcal isolates in this study is available at https://microreact.org/project/lebanon-gps. The authors confirm that all supporting data, code and protocols have been provided within the article or through supplementary data files.

## Introduction

*Streptococcus pneumoniae* is an opportunistic pathogen that can cause invasive pneumococcal diseases (IPDs), including pneumonia, meningitis and sepsis [1]. In 2015, IPD was estimated to have caused 294,000 deaths among children aged 1 through 59 months [2]. The burden of IPD is also high amongst older adults, largely due to comorbidities and age-related waning of the immune system [3]. The incidence rate and case-fatality ratio of IPD are higher in low-income countries like Bangladesh, Nepal and Gambia, compared to upper-middle income and high-income countries [4]. Although pneumococcal infections are treatable with antimicrobials, the outcome is not always favourable due to the rapid progression of invasive infections and extensive tissue damage [5]. In 2021, a global analysis identified *S. pneumoniae* as one of the top three pathogens responsible for the highest number of deaths attributable to antimicrobial resistance (AMR) among children younger than 5 years. The study also reported a rise in AMR-related deaths caused by *S. pneumoniae* among people aged 5 years and older between 1990 and 2021 [6].

Vaccination remains a cornerstone in the prevention of IPD. Between 2000 and 2015, the introduction of pneumococcal conjugate vaccines (PCVs) has contributed to a 51% global reduction in deaths linked to pneumococcal diseases, despite each PCV formulation only containing a limited range of serotypes [2]. PCV, including the 7-valent PCV (PCV7) and the 13-valent PCV (PCV13), is effective in reducing IPD caused by the serotypes present in their formulation [7,8]. However, the selective pressure exerted by PCV has altered the composition of *S. pneumoniae* populations, leading to serotype replacement and the expansion of non-vaccine type (NVT) strains [9,11]. Interestingly, the impact of serotype replacement varies geographically based on factors such as vaccine uptake rates and the baseline pneumococcal population [12]. PCVs also directly and indirectly reduce the prevalence of AMR in pneumococci. They directly reduced the prevalence of highly resistant pneumococcal strains and also reduced the circulation of *S. pneumoniae* in children and adults, leading to fewer infections that require antimicrobial treatment [13].

In Lebanon, PCV7 and PCV13 were introduced in 2006 and 2010, respectively, into the private healthcare sector [14], where about half of the child population received their vaccinations. In addition to the private sector, PCV13 was incorporated into the national Expanded Program on Immunization (EPI) in January 2016, extending vaccine coverage to the public healthcare sector, where it was administered until the end of 2024 using a 2+1 schedule at 4, 6 and 12 months of age. An adult pneumococcal vaccination programme has not yet been implemented in Lebanon. Adult vaccination remains at the discretion of the treating physician. The primary aim of this study was to characterize invasive *S. pneumoniae* isolates collected from different regions across Lebanon using whole-genome sequencing (WGS). The isolates were sampled from an ongoing active surveillance programme during the Private-PCV7 (2003–2009), Private-PCV13 (2010–2015) and EPI-PCV13 (2016–2025) periods. Additionally, we assessed the impact of PCV introduction on the circulating pneumococcal serotypes, lineages and AMR profiles. Finally, we investigated the association between vaccine and non-vaccine types with IPD clinical manifestations.

## Methodology

### Study design

The *S. pneumoniae* isolates (*n*=277) included in this multicentre study were collected between April 2003 and January 2025 as part of the Lebanese Inter-Hospital Pneumococcal Surveillance Program (LIPSP) (Fig. S1, available in the online Supplementary Material). LIPSP is an active surveillance programme that conducts epidemiological and genomic surveillance of invasive *S. pneumoniae* isolates in Lebanon. LIPSP was established by the Center for Infectious Diseases Research (CIDR) at the American University of Beirut, in collaboration with the Lebanese Ministry of Public Health. Sequencing was performed for the *S. pneumoniae* isolates collected, of which 273 passed quality control (QC) and were analysed further.

### Definitions

As per the Centers for Disease Control and Prevention, IPD was defined as the isolation of *S. pneumoniae* from a normally sterile body site [15]. Regarding pneumococcal serotype classification, PCV7 refers to isolates with serotypes 4, 6B, 9V, 14, 18C, 19F and 23F. PCV13-only refers to the additional six serotypes not included in PCV7, namely 1, 3, 5, 6A, 7F and 19A. NVT refers to all other non-PCV13 serotypes. During the study period, PCV7 and PCV13 were available in Lebanon.

The study periods were defined based on the introduction of PCV in Lebanon. The Private-PCV7 period refers to the period from 2003 to 2009, when the PCV7 vaccine was in use only in the private healthcare sector. The Private-PCV13 period spans from 2010 to 2015, following the addition of PCV13 to the private healthcare sector, but before its incorporation into EPI. The EPI-PCV13 period covers the period from 2016 to 2025, after PCV13 was introduced into the EPI, becoming available in the public healthcare sector alongside continued administration in the private healthcare sector.

To assess vaccine coverage across the different study periods, we calculated the serotype coverage for PCV7 (Pfizer), PCV13 (Pfizer), PCV10 (Pneumosil), PCV15 (Merck), PCV20 (Pfizer) and PCV24 (GSK) (Table 1) based on the serotype composition of each vaccine. The detailed vaccine formulations are available at https://www.pneumogen.net/gps/#/about#outline.

**Table 1.** PCV coverage of invasive pneumococcal isolates from Lebanon between 2003 and 2025 according to study period (*n*=272) or clinical manifestation (*n*=273). Coverage was calculated based on the serotype composition of each vaccine

		No. of isolates (%)					
	n	PCV7 (Pfizer)	PCV10 (Pneumosil)	PCV13 (Pfizer)	PCV15 (Merck)	PCV20 (Pfizer)	PCV24 (GSK)[Table-fn T1_FN1]*
Study period							
Private-PCV7	30	24 (80.0%)	29 (96.6%)	29 (96.6%)	29 (96.6%)	30 (100.0%)	30 (100.0%)
Private-PCV13	86	39 (45.3%)	49 (56.9%)	59 (68.6%)	62 (72.0%)	65 (75.5%)	66 (76.7%)
EPI-PCV13	156	28 (17.9%)	42 (26.9%)	78 (50%)	84 (53.8%)	106 (67.9%)	113 (72.4%)
Total	272	91 (33.4%)	120 (44.1%)	166 (61.0%)	175 (64.3%)	201 (73.8%)	209 (76.8%)
Clinical manifestation							
Pneumonia	123	37 (30.0%)	60 (48.7%)	82 (66.6%)	87 (70.7%)	96 (78.0%)	98 (79.6%)
Bacteraemia	95	33 (34.7%)	39 (41.0%)	54 (56.8%)	57 (60%)	69 (72.6%)	72 (75.7%)
Meningitis	38	17 (44.7%)	17 (44.7%)	24 (63.1%)	25 (65.7%)	27 (71.0%)	29 (76.3%)
Other/unknown	17	4 (23.5%)	5 (29.4%)	7 (41.1%)	7 (41.1%)	10 (58.8%)	11 (64.7%)
Total	273	91 (33.3%)	121 (44.3%)	167 (61.1%)	176 (64.4%)	202 (73.9%)	210 (76.9%)

* Investigational PCV.

Finally, an isolate was designated as multidrug-resistant (MDR) if it was non-susceptible to at least three of the following antibiotics: penicillin (PEN), erythromycin (ERY), tetracycline (TET), cotrimoxazole (COT) and chloramphenicol (CHL). The MICs of PEN and ceftriaxone (CFT) were inferred from genomic data and interpreted based on the meningitis cut-off outlined in the Clinical Laboratory Standards Institute (CLSI) guidelines [16]. Hence, PEN and CFT with MICs≤0.06 µg ml^−1^ and ≤0.5 µg ml^−1^, respectively, were categorized as susceptible.

### Target population and study site

Specimens were obtained from patients hospitalized with IPD and admitted to 35 different hospitals, covering all regions of the country. *S. pneumoniae* was isolated from blood (*n*=235), cerebrospinal fluid (*n*=28), body fluid (*n*=9) and urine (*n*=1). The urine sample was obtained from a patient with pyelonephritis that originated from a bloodstream infection. The study included patients of all age groups and both sexes. For each sample, de-identified epidemiological and clinical data were collected, including sex, age, comorbidities, clinical syndrome and outcome. Patients were followed up until hospital discharge, with mortality referring only to in-hospital deaths.

### Bacterial identification and sample processing

Colony morphology and optochin susceptibility were used for *S. pneumoniae* identification. Upon receipt at the CIDR laboratory, pneumococcal isolates were stored at −80 °C in sterile 2 ml cryogenic vials for further processing. The frozen isolates were subject to WGS at either the Wellcome Sanger Institute (*n*=214) or locally at the CIDR laboratory (*n*=63).

At the CIDR laboratory, dsDNA was extracted using the Quick-DNA^™^ HMW MagBead Kit (Zymo Research, USA) according to the manufacturer’s instructions. DNA was quantified using the Qubit fluorometer (Life Technologies, Carlsbad, CA, US) with the High Sensitivity dsDNA Assay Kit. At the Wellcome Sanger Institute, DNA was purified using the QIAcube HT instrument and QIAamp 96 DNA QIAcube HT Kit (Qiagen, Germany) according to the manufacturer’s instructions.

### Whole-genome sequencing

WGS was performed using the Illumina NovaSeq platform at the Wellcome Sanger Institute (*n*=214) or using the Illumina MiSeq (*n*=51) and Oxford Nanopore Minion (*n*=12) platforms at CIDR. The NEBNext^®^ Ultra^™^ II DNA Library Prep Kit for Illumina and the Illumina NovaSeq 6000 system (Illumina, San Diego, CA) were utilized at the Wellcome Sanger Institute for sequencing. At CIDR, Illumina libraries were prepared using the Illumina DNA prep kit (Illumina, San Diego, CA) per the manufacturer’s protocol. The MiSeq V2 Reagent Kit was used for sequencing the pooled DNA library on the Illumina MiSeq platform (Illumina, San Diego, CA).

For long-read sequencing, the DNA library was prepared using the Oxford Nanopore Rapid Barcoding Kit 96 V14 (SQK-RBK114.96) following the manufacturer’s protocol. The library was loaded into an R10.4.1 flow cell (FLO-MIN114) and sequenced using the MinION Mk1C device (Oxford Nanopore Technologies, UK).

### Genomic analysis

Illumina FastQ files (*n*=265) were processed using the GPS pipeline (v1.0.0-rc12) with default parameters [17] to infer *in silico* serotype, pneumococcal lineages (multilocus sequence types, ST; global pneumococcal sequence clusters, GPSCs), pili virulence factors and AMR from the genomic data. AMR analysis was performed for 19 antibiotics, including PEN, amoxicillin (AMO), meropenem, cefotaxime, CFT, cefuroxime (CFX), ERY, clindamycin (CLI), sulphamethoxazole, trimethoprim, doxycycline, COT, TET, kanamycin, levofloxacin, CHL, fluoroquinolones (FQ), rifampin and vancomycin. For beta lactam antibiotics, MIC values were predicted by the GPS pipeline based on a PEN-binding protein (PBP) typing method developed by Li *et al*., which assigns a PBP type based on a signature sequence in the transpeptidase domain of three key PBPs, namely PBP1a, PBP1b and PBP2x [18,19]. The predicted MIC values were interpreted according to CLSI breakpoints [16]. For the remaining antibiotics, resistance was predicted based on the absence or presence of genomic resistance markers as previously reported [17]. High concordance between the phenotypic and genotypic results for serotypes and antibiotic resistance was previously reported [20].

FastQ files from long-read sequencing (*n*=12) were analysed using the assemble-BAC-ONT pipeline (v1.1) [21] to generate polished FASTA files using Flye (v2.9-b1768) and medaka (v1.4.4). The assembled ONT (Oxford Nanopore Technologies) FASTA files were then converted into paired-end reads using fastaq (v3.17.0). The resulting Fastq files were subsequently analysed using the GPS pipeline (v1.0.0-rc12).

Phylogenetic analysis was performed by constructing a maximum-likelihood tree for the isolates based on SNPs using FastTree (v2.1.1) [22]. The SNP alignment was derived from an alignment generated by mapping reads to the *S. pneumoniae* ATCC700669 reference genome (NCBI accession number FM211187) using snippy (v4.6.0) (https://github.com/tseemann/snippy) and SNP-sites (v2.5.1) [23].

### Statistical analysis

For categorical variables, data were described as frequency and percentage [n, (%)] and analysed using GraphPad (v8.4) for Windows (GraphPad Software, La Jolla, CA, USA). The chi-square test (χ² test) was used for the analysis of categorical variables when the frequency in each cell was ≥5. Otherwise, Fisher’s exact test was used when the frequency in each cell was <5. These tests were used to determine if significant differences were observed in the prevalence of AMR and MDR pneumococci between age groups. Moreover, they were utilized to investigate the association between pili detection and MDR and to examine the prevalence of clinical syndromes according to vaccine and non-vaccine types. *P*-values were two-sided, with statistical significance set at *P*-value<0.05. Visualizations were created using the Tidyverse package (v2.0.0) in R, and all code is available at https://github.com/ak2022/Lebanon.

## Results

### Demographic and clinical characteristics

Of 277 *S. pneumoniae* isolates, 273 were successfully sequenced and passed the genome QC parameters of the GPS pipeline. The isolates were obtained from Beirut (*n*=166), North Lebanon (*n*=69), Mount Lebanon (*n*=22), South Lebanon (*n*=12) and Bekaa (*n*=4). Of the patients with available sex data, more than half were males (*n*=153/266; 57.5%). A total of 268 patients had known age data. Of these, 101 patients (37.6%) were aged 5 years or younger, 81 patients (30.2%) were between 6 and 60 years, and 86 patients (32.0%) were above 60 years. The three most common clinical syndromes in all IPD patients in this study were pneumonia (*n*=123, 45.0%), bacteraemia (*n*=95, 34.7%) and meningitis (*n*=38, 13.9%).

### Serotypes and lineages causing invasive disease

**Fig. 1.**
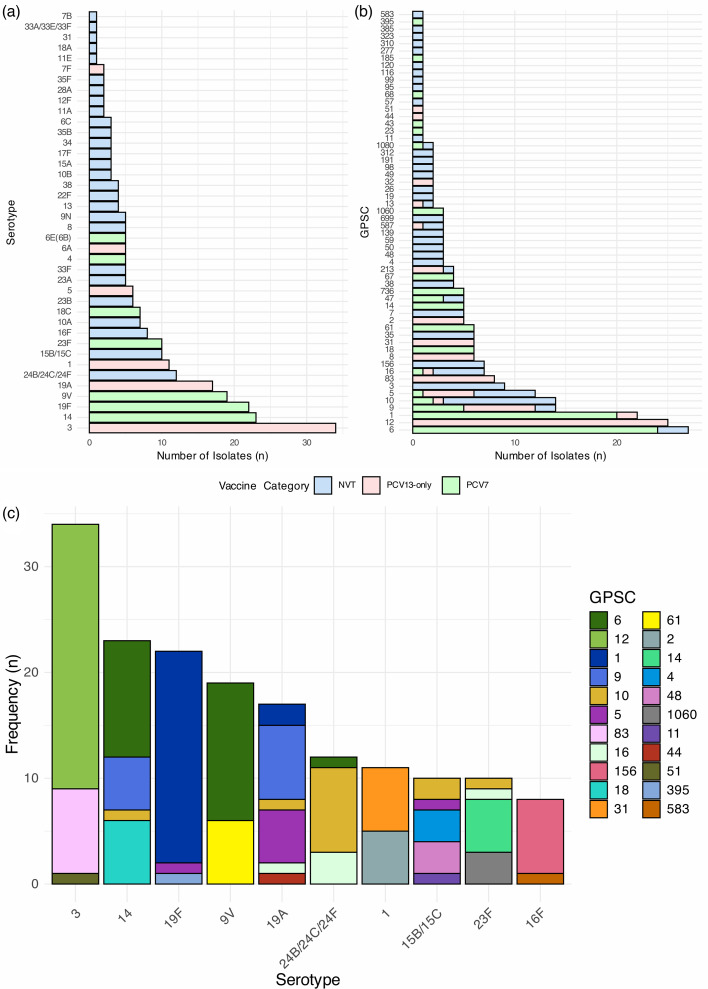
Serotype (**a**) and GPSC (**b**) distribution of the *S. pneumoniae* isolates collected from IPD patients in Lebanon between 2003 and 2025. Green bars represent serotypes included within PCV7, pink bars represent the additional serotypes within PCV13, and blue bars represent NVT serotypes. (**c**) GPSC distribution among the 10 most prevalent *S. pneumoniae* serotypes identified in Lebanon (3, 14, 19F, 9V, 19A, 24B/24C/24F, 1, 15B/15C, 23F and 16F; *n*=166). Abbreviations: GPSC, global pneumococcal sequence clusters; PCV7 refers to serotypes covered by the 7-valent PCV (PCV7); PCV13 represents additional serotypes covered by the 13-valent PCV (PCV13), but not PCV7; NVTs are serotypes that are not covered by PCV13.

A total of 40 serotypes were predicted from WGS data (Fig. 1a). Overall, serotype 3 (*n*=34/273; 12.5%) was the most prevalent, followed by serotypes 14 (*n*=23/273; 8.4%), 19F (*n*=22/273; 8.1%) and 9V (*n*=19/273; 7.0%). A total of 58 GPSCs were identified, encompassing 97 different STs. The most predominant pneumococcal lineages were GPSC6 (*n*=27/273; 9.9%), GPSC12 (*n*=25/273; 9.2%) and GPSC1 (*n*=22/273; 8.1%), which together accounted for 27.1% of isolates (Fig. 1b).

PCV7 serotypes accounted for 33.3% (*n*=91/273) of all isolates, with serotypes 14, 19F and 9V being the most prevalent (Fig. 1a). Serotype 19F was expressed by GPSC1 (*n*=20/22; 90.9%), GPSC5 (*n*=1/22, 4.5%) and GPSC395 (*n*=1/22, 4.5%) (Fig. 1c). Serotype 9V was mostly expressed by GPSC6 (*n*=13/19; 68.4%) followed by GPSC61 (*n*=6/19; 31.6%). Serotype 14 was expressed by GPSC6 (*n*=11/23; 47.8%), GPSC18 (*n*=6/23; 26.1%), GPSC9 (*n*=5/23; 21.7%) and GPSC10 (*n*=1/23; 4.3%). Among the 273 isolates, PCV13-only serotypes accounted for 27.8% (*n*=76/273) of the total serotypes, with serotypes 3, 19A and 1 being the most prevalent (Fig. 1a). Serotype 3 (*n*=34/76; 44.7%) was expressed by GPSC12 (*n*=25/34; 73.5%), followed by GPSC83 (*n*=8/34; 23.5%) and GPSC51 (*n*=1/34; 2.9%) (Fig. 1c). Serotype 19A (*n*=18/76; 23.7%) and serotype 1 (*n*=12/76; 15.8%) were mostly expressed by GPSC9 (*n*=8/18; 44.4%) and GPSC31 (*n*=6/11; 54.5%), respectively.

NVT constituted 38.8% (*n*=106/273) of all *S. pneumoniae* isolates analysed in this study. Among these, serotypes 24B/24C/24F (*n*=12/106; 11.3%), 15B/15C (*n*=10/106; 9.4%) and 16F (*n*=8/106; 7.5%) were the most prevalent (Fig. 1a). Serotype 24B/24C/24F was expressed by GPSC10 (*n*=8/12; 66.7%), GPSC16 (*n*=3/12; 25.0%) and GPSC6 (*n*=1/12; 8.3%) isolates (Fig. 1c). Serotype 15B/15C was expressed by GPSC4 (*n*=3/10; 30.0%), GPSC48 (*n*=3/10; 30.0%), GPSC10 (*n*=2/10; 20.0%), GPSC11 (*n*=1/10; 10.0%) and GPSC5 (*n*=1/10; 10.0%). Serotype 16F was mostly expressed by GPSC156 (*n*=7/8; 87.5%).

### Serotypes and lineages by vaccine period

**Fig. 2.**
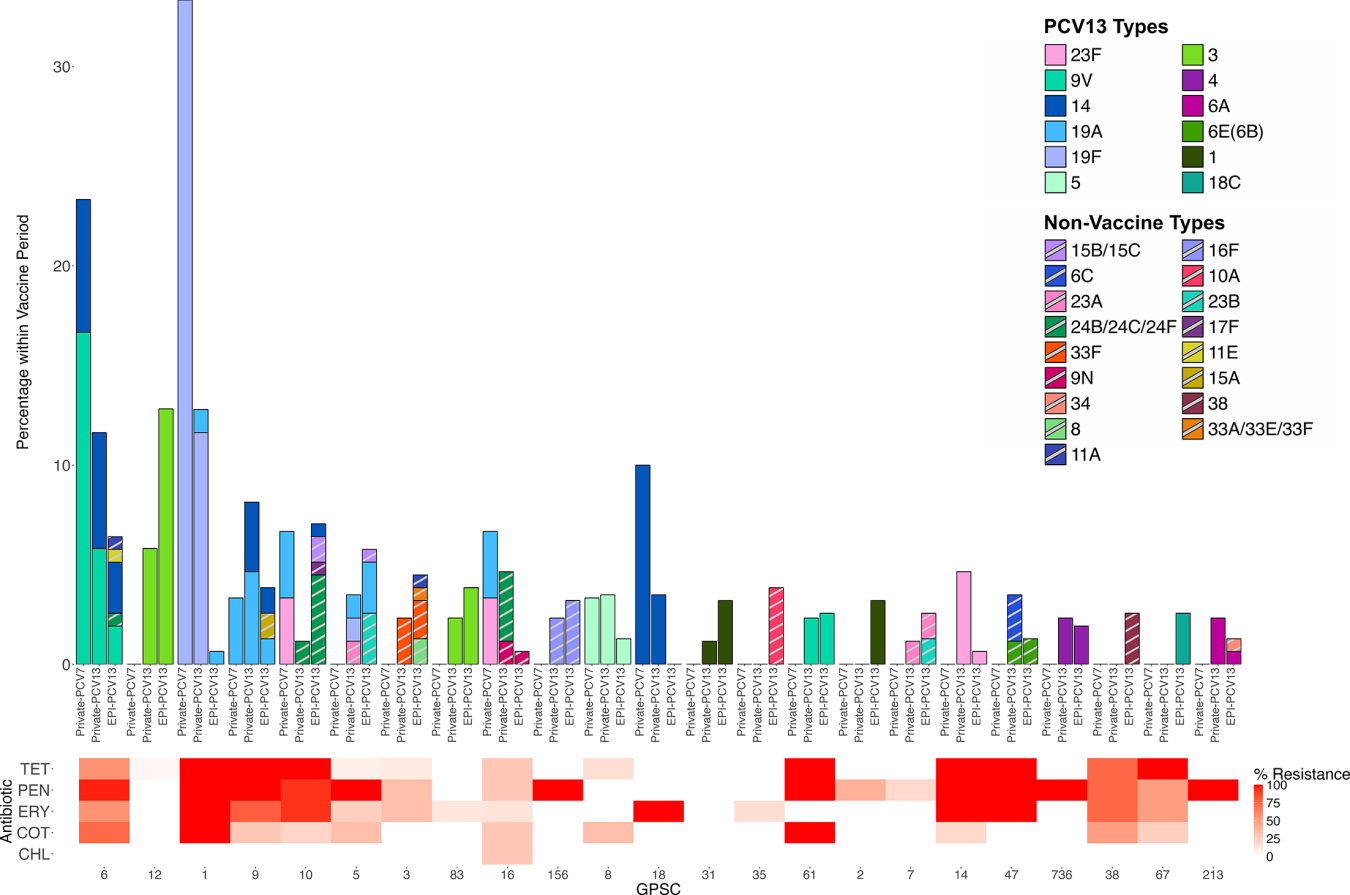
Distribution of GPSC and serotypes within the collection over time, alongside prevalence of antibiotic resistance. Only GPSCs with counts of four or greater in the dataset were included. Many commonly seen lineages are MDR, including GPSC1, GPSC6 and GPSC9. This figure includes 26 genomes from the Private-PCV7 period, 68 genomes from the Private-PCV13 period and 118 genomes from the EPI-PCV13 period. Non-PCV13 serotypes are denoted with stripes. Abbreviations: CHL, chloramphenicol; COT, cotrimoxazole; ERY: erythromycin; GPSC, global pneumococcal sequence clusters; PEN: penicillin; TET: tetracycline.

The genomes analysed in this study included 30 (11.0%) from the Private-PCV7 period, 86 (31.6%) from the Private-PCV13 period and 156 (57.4%) from the EPI-PCV13 period. One isolate had no year of collection data and was excluded from this analysis. To gain a deeper understanding of the current disease-causing pneumococcal population in Lebanon, we investigated the circulating lineages and serotypes within the most recent vaccine period, the EPI-PCV13 period. The most prevalent serotypes in this period were serotype 3 (*n*=26/156; 16.7%) and serotype 1 (*n*=10/156; 6.4%) (Fig. 2). Of the 156 isolates collected within this timeframe, 50.0% were NVT (*n*=78/156), of which serotypes 24B/24C/24F (*n*=8/156; 5.1%), 15B/15C (*n*=8/156; 5.1%) and 10A (*n*=7/156; 4.5%) were the most prevalent. None of these serotypes are included within the PCV15 formulation, and only serotypes 10A and 15B are included within the PCV20 formulation. The most prevalent lineages in the EPI-PCV13 period were GPSC12 (*n*=20/156; 12.8%), GPSC10 (*n*=11/156; 7.1%) and GPSC6 (*n*=10/156; 6.4%) (Fig. 2). GPSC12 only consisted of serotype 3 within this period, whereas GPSC10 contained NVT serotypes 24B/24C/24F (*n*=7/11; 63.6%), 15B/15C (*n*=2/11; 18.2%), 14 (*n*=1/11; 9.1%) and 17F (*n*=1/11; 9.1%). GPSC6 contained serotypes 14 (*n*=4/10; 40.0%), 9V (*n*=3/10; 30.0%), 11A, 11E and 24B/24C/24F (*n*=1/10; 10.0% each). Whilst both serotypes 14 and 9V are PCV7 serotypes, the others are NVT.

**Fig. 3.**
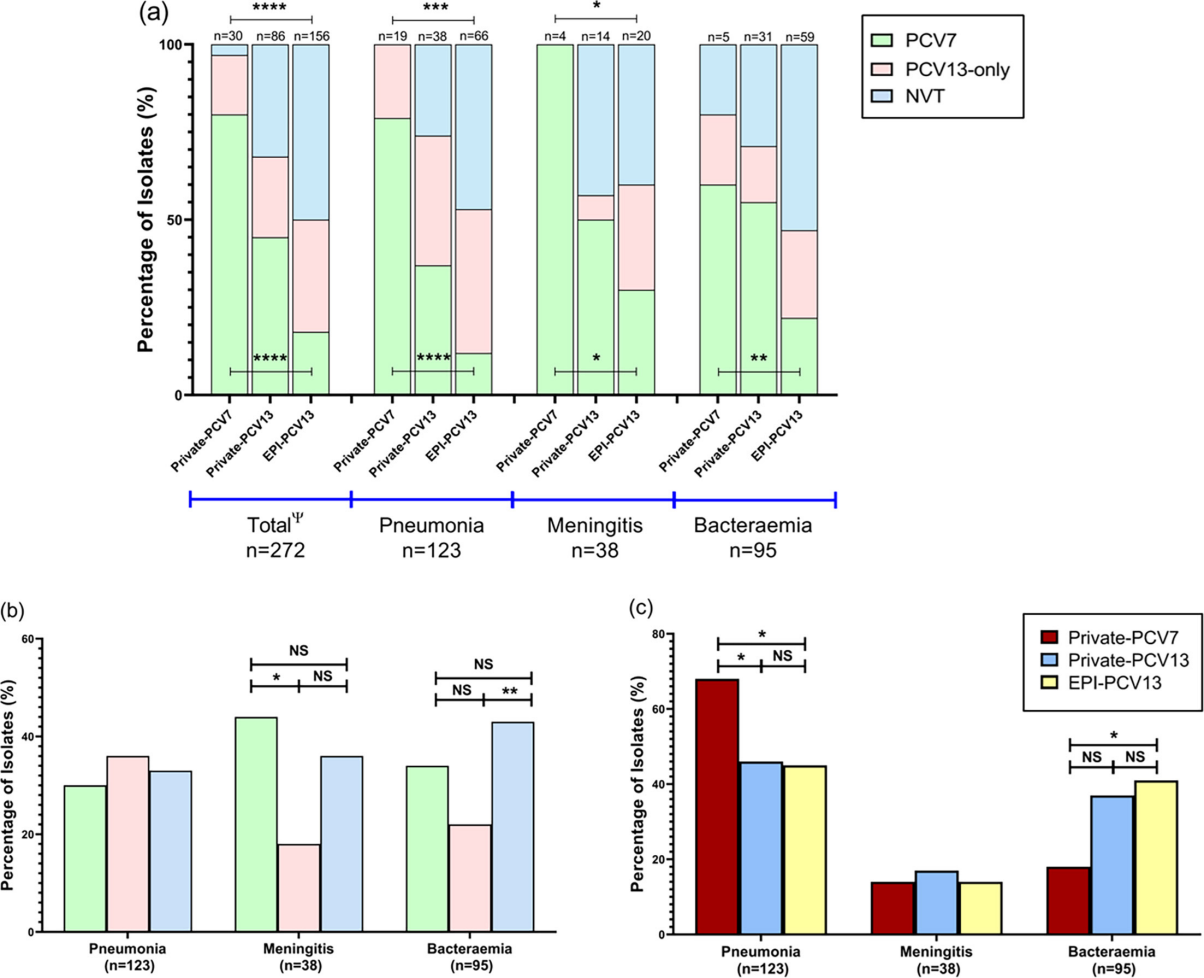
The distribution of vaccine serotypes and NVTs across the three study periods by clinical syndrome (**a**). Prevalence of pneumonia, bacteremia and meningitis in invasive *S. pneumoniae* isolates according to vaccine and non-vaccine serotypes (**b**). Prevalence of pneumonia, bacteremia and meningitis in invasive *S. pneumoniae* isolates in each vaccine period (**c**). Significant differences were calculated using the Chi Square or Fisher’s exact tests. Significant values are indicated with *(*P*<0.05), **(*P*<0.01), ***(*P*<0.001) or ****(*P*<0.0001). Non-significant values are indicated with NS. ‘Ψ One isolate had missing collection date. In panel (a), asterisks above the bars indicate statistically significant differences in NVT proportions across periods, while asterisks below the bars indicate significant differences in PCV7 proportions. No statistically significant differences were observed when comparing PCV13-only serotypes across periods. Significance was assessed using the Chi-Square test across three groups (Private-PCV7, Private-PCV-13 and EPI-PCV13 periods). The study includes 30 genomes from the Private-PCV7 period, 86 genomes from the Private-PCV13 period and 156 genomes from the EPI-PCV13 period. Abbreviations: PCV7 refer to serotypes covered by the 7-valent pneumococcal conjugate vaccine (PCV7). PCV13-only represent additional serotypes covered by the 13-valent pneumococcal conjugate vaccine (PCV13), but not PCV7. NVT are serotypes that are not covered by PCV13.

We investigated changes in the prevalence of vaccine types (VTs) and NVT following vaccine introduction. PCV7 serotypes accounted for 80.0% of isolates (*n*=24/30) in the Private-PCV7 period but declined significantly after vaccine introduction, reaching 45.3% (*n*=39/86) in the Private-PCV13 (*P*<0.0001) (Fig. 3a). PCV7 serotypes further declined to 17.9% (*n*=28/156) in the EPI-PCV13 (*P*<0.0001). In contrast, the prevalence of NVT increased significantly from 3.3% (*n*=1/30) during the Private-PCV7 period to 31.3% (*n*=20/86) in the Private-PCV13 period (*P*<0.0001). We detected a further significant increase in the prevalence of NVT over time, which reached 50.0% (*n*=78/156) in the EPI-PCV13 (*P*<0.0001). Compared to the more pronounced shifts in NVT and PCV7 serotypes across the vaccine periods, PCV13-only serotypes exhibited more gradual increases. The prevalence of PCV13-only serotypes was 16.6% (*n*=5/30) in the Private-PCV7 period, 23.2% (*n*=20/86) in the Private-PCV13 period and 32.1% (*n*=50/156) in the EPI-PCV13 period.

### Serotypes by clinical manifestations and mortality outcome

The prevalence of PCV7 serotypes in pneumonia was significantly higher in the Private-PCV7 period (*n*=15/19; 78.9%) compared to the Private-PCV13 (*n*=14/38; 36.8%) and EPI-PCV13 (*n*=8/66; 12.12%) periods (*P*<0.0001) (Fig. 3a). NVTs were not detected among the genomes associated with pneumonia during the Private-PCV7 period. However, the prevalence of NVT causing pneumonia significantly increased from 26.3% in the Private-PCV13 period (*n*=10/38) to 46.9% in the EPI-PCV13 period (*n*=31/66) (*P*=0.0004). All isolates from patients with meningitis were PCV7 serotypes in the Private-PCV7 period (*n*=4; 100.0%), but this decreased significantly to 50.0% in the Private-PCV13 period (*n*=7/14) and 30.0% in the EPI-PCV13 (*n*=6/20) period (*P*=0.0325) (Fig. 3a). Moreover, NVTs causing meningitis increased from none during the Private-PCV7 period (*n*=0/4) to 40.0% in the EPI-PCV13 period (*n*=8/20) (*P*=0.0169). The prevalence of PCV7 serotypes decreased significantly in bacteraemia cases from 60% (*n*=3/5) during the Private-PCV7 period to 22.0% (*n*=13/59) in the EPI-PCV13 period (*P*=0.0038).

There was no significant association between pneumonia and PCV7, PCV13-only or NVT pneumococcal serotypes (Fig. 3b). PCV7 serotypes accounted for 44.7% (*n*=17/38) of meningitis cases in the dataset, constituting a significantly higher proportion compared to PCV13-only serotypes (*n*=7/38; 18.4%) (*P*=0.0136). Furthermore, NVTs were significantly more prevalent in bacteraemia cases (*n*=41/95; 43.1%) compared to PCV13-only serotypes (*n*=21/95; 22.1%) (*P*=0.002). We examined the trends in IPD clinical manifestations across the different vaccine periods in this study (Fig. 3c). During the Private-PCV7 period, pneumonia constituted 67.8% (*n*=19/28) of IPD clinical manifestations, which was significantly higher compared to the Private-PCV13 (*n*=38/83; 45.7%; *P*=0.0433) and EPI-PCV13 periods (*n*=66/145; 45.5%; *P*=0.0304) (Fig. 3c). In the EPI-PCV13 period, bacteraemia constituted a higher proportion of IPD manifestations (*n*=59/145; 40.6%) compared to the Private-PCV7 period (*n*=5/28; 17.8%) (*P*=0.022).

**Fig. 4.**
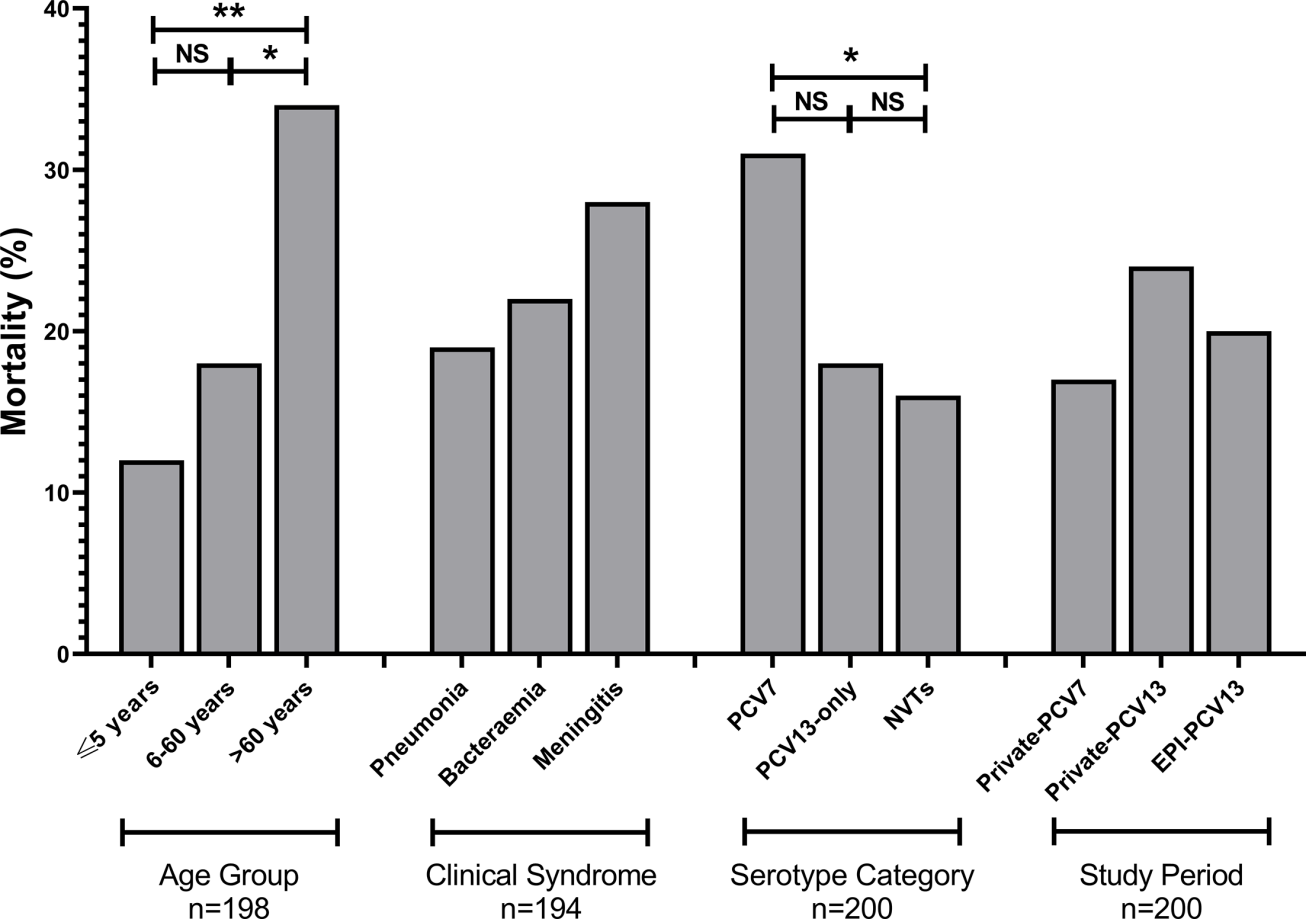
Mortality rates across different age groups, pneumococcal serotypes, clinical manifestations and study periods. Mortality outcomes were recorded for 200 IPD patients. Significant differences were calculated using the Chi Square or Fisher’s exact tests. Significant values are indicated with *(*P*<0.05) or **(*P*<0.01). Abbreviations: PCV7 refer to serotypes covered by the 7-valent pneumococcal conjugate vaccine (PCV7); PCV13 represent additional serotypes covered by the 13-valent pneumococcal conjugate vaccine (PCV13), but not PCV7; NVT are serotypes that are not covered by PCV13.

Out of the 273 patients included in the analysis, 200 had a documented mortality outcome. Differences in mortality rates between age groups are shown in Fig. 4. The mortality rate was significantly higher among patients aged over 60 years (*n*=21/61; 34.4%) compared to those aged ≤5 years (*n*=9/76; 11.8%; *P*=0.0015) and those aged 6–60 years (*n*=11/61; 18.0%; *P*=0.0396). Furthermore, PCV7 serotypes exhibited significantly higher mortality (*n*=19/61; 31.1%) compared to NVTs (*n*=14/88; 15.9%) (*P*=0.0276). There were no significant differences in mortality rates between the different IPD clinical manifestations or vaccine periods.

### PCV coverage by study period and clinical manifestations

Overall, the projected serotype coverage for PCV7 (Pfizer), PCV10 (Pneumosil), PCV13 (Pfizer), PCV15 (Merck) and PCV20 (Pfizer) was 33.3% (*n*=91/273), 44.3% (*n*=121/273), 61.1% (*n*=167/273), 64.4% (*n*=176/273) and 73.9% (*n*=202/273), respectively (Table 1). Among PCVs under development, the projected coverage was 76.9% (*n*=210/273) for PCV24 (GSK). When analysed by study period (Table 1), PCV7 coverage was highest during the Private-PCV7 period (*n*=24/30; 80.0%), declining to 45.3% (*n*=39/86) during the Private-PCV13 period and further to 17.9% (*n*=28/156) in the EPI-PCV13 period. During the EPI-PCV13 period (*n*=156), which represents the most recent vaccine period in this study, serotype coverage was 50.0% (*n*=78/156) for PCV13, which is around double that of PCV10 (*n*=42/56; 26.9%). Coverage by PCV15 was only slightly higher at 53.8% (*n*=84/156), as the additional PCV15 serotypes 22F and 33F were seen infrequently in the dataset (*n*=6/156). PCV20 is likely to provide improved serotype coverage (*n*=106/156; 67.9%) as it includes the first and third most frequently seen NVTs in the EPI-PCV13 time period (15B, *n*=8/156; 10A, *n*=7/156).

Notably, PCV10 (Pneumosil) would offer lower coverage across all clinical manifestations compared to PCV13 (Pfizer). Among licensed PCV, PCV20 (Pfizer) demonstrated the highest coverage overall (73.9%), and across all clinical manifestations. The highest PCV coverage in pneumonia (79.6%), bacteraemia (75.7%) and meningitis (76.3%) was obtained with PCV24 (GSK), an investigational PCV.

### AMR profiles

Overall, non-susceptibility to PEN (*n*=170/273; 62.2%) was the most prevalent AMR detected among the study isolates, followed by COT (*n*=142/269; 52.7%) and TET (*n*=109/273; 39.9%) (Table S1, available in the online Supplementary Material). The GPS pipeline reported COT AMR as indeterminable in four isolates. Non-susceptibility to ERY, CLI and CFT was predicted in 38.4% (*n*=105/273), 26.3% (*n*=72/273) and 24.9% (*n*=68/273) of genomes, respectively. Non-susceptibility to CHL (*n*=3/273; 1.0%) and FQ (*n*=1/273; 0.3%) was rare. MDR, defined as non-susceptibility to three of the following antibiotics: PEN, CHL, COT, ERY and TET, was identified in 36.6% (*n*=100/273) of isolates. Notably, the prevalence of MDR was significantly higher in PCV7 serotypes (*n*=62/91; 68.1%) compared to PCV13-only serotypes (*n*=15/76; 19.7%) and NVTs (*n*=23/106; 21.6%) (*P*<0.0001) (Table S1).

**Fig. 5.**
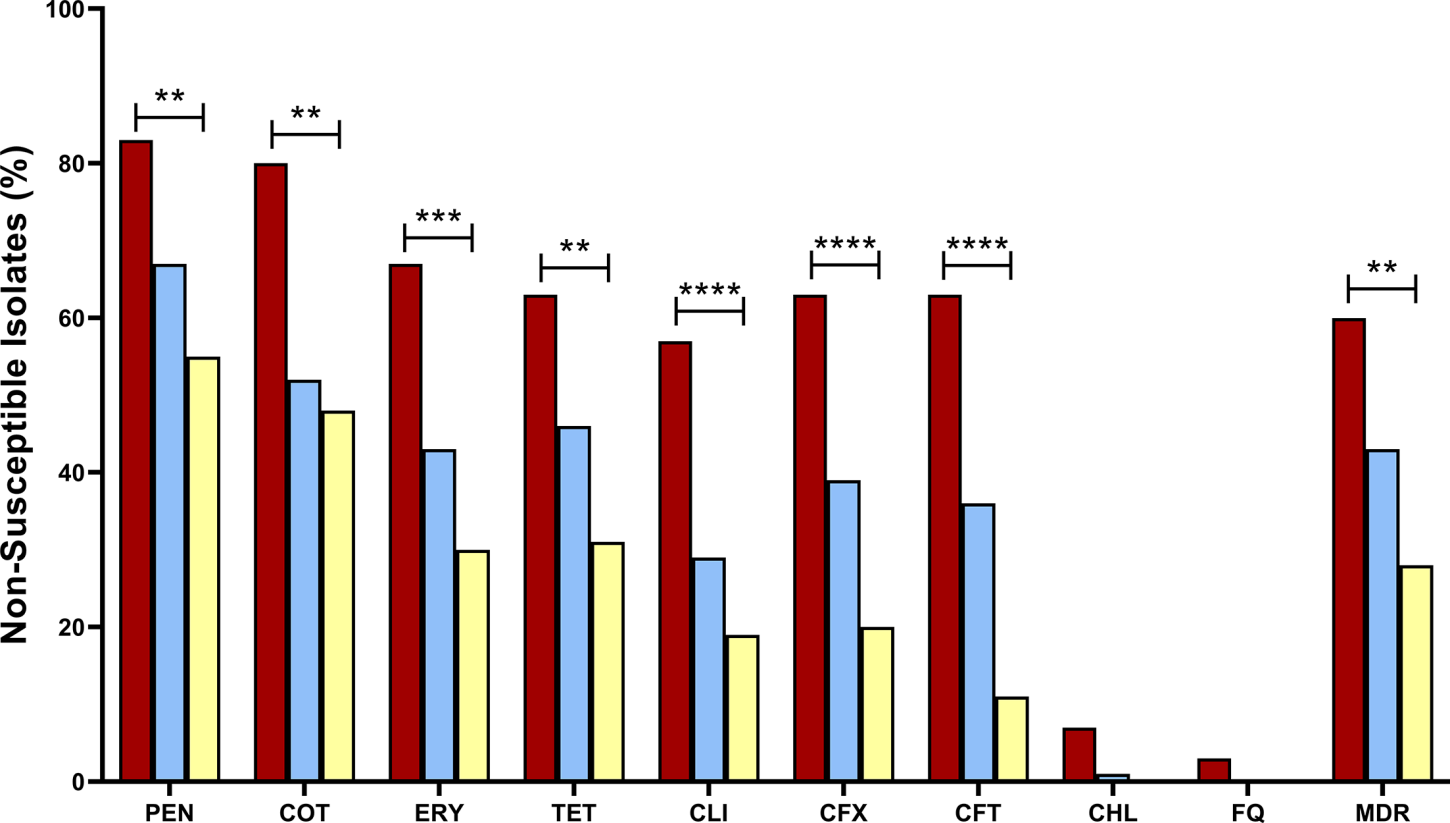
Trends in antibiotic non-susceptibility among *S. pneumoniae* (*n*=272) over the three vaccine periods. Significant differences were calculated using the chi-square test across three groups to show significant changes across the periods. Significant values are indicated with **(*P*<0.01), ***(*P*<0.001) or ****(*P*<0.0001). The year of collection was missing for one isolate, and COT AMR was indeterminable in four isolates. Abbreviations: CFT, ceftriaxone; CFX, cefuroxime; CHL, chloramphenicol; CLI, clindamycin; COT, cotrimoxazole; ERY, erythromycin; FQ, fluoroquinolones; MDR, multidrug resistant; PEN, penicillin; TET, tetracycline.

The overall prevalence of AMR and MDR decreased significantly after PCV introduction. During the Private-PCV7 period, non-susceptibility prevalence was highest for PEN (*n*=25/30; 83.3%), COT (*n*=24/30; 80.0%) and ERY (*n*=20/30; 66.7%), and 60.0% (*n*=18/30) of genomes were classified as MDR (Fig. 5).

In the Private-PCV13 period, a significant decreasing trend in non-susceptibility rates to seven antibiotics was detected, including PEN (*n*=58/86; 67.4%) and COT (*n*=44/84; 52.3%). Moreover, MDR prevalence was 43.0% (*n*=37/86) in the Private-PCV13 period. In the EPI-PCV13 period, a significant decline in non-susceptibility was observed across seven antibiotics, with CFT resistance dropping to 11.5% (*n*=18/156), ERY to 30.1% (*n*=47/156) and TET to 31.4% (*n*=49/156). MDR prevalence was 28.2% (*n*=44/156) in the EPI-PCV13 period. Over half of the isolates were predicted to be non-susceptible to PEN (*n*=86/156; 55.1%) in the EPI-PCV13 period.

Among NVTs, only non-susceptibility to COT was significantly higher in the EPI-PCV13 period (*n*=38/76; 50.0%) compared to the Private-PCV13 period (*n*=7/26; 26.9%) (*P*=0.0408) (Table S2). No other significant changes in AMR were seen in the NVT population specifically.

The prevalence of non-susceptibility to PEN, TET, ERY and CLI was similar between all age groups (Fig. S2A). Non-susceptibility to COT was significantly higher in infants (*n*=60/101; 59.4%) compared to older adult patients (*n*=36/84; 42.8%) (*P*=0.0249). CFT non-susceptibility among patients aged ≤5 years was significantly reduced in the EPI-PCV13 period (*n*=6/59; 10.1%) compared to the Private-PCV7 (*n*=10/15; 66.6%; *P*<0.0001) and Private-PCV13 periods (*n*=12/26; 46.1%; *P*=0.0002) (Fig. S2B). Similarly, in the ≤5 years age group, ERY, CLI and CFX non-susceptibility exhibited a significant decrease over the study periods. The proportion of MDR in patients aged 6–60 years significantly decreased from 83.3% (*n*=5/6) in the Private-PCV7 period to 23.2% (*n*=10/43) in the EPI-PCV13 period (*P*=0.0077) (Fig. S2C). Moreover, non-susceptibility to COT, CLI, TET and CFT was significantly lower during the EPI-PCV13 compared to the Private-PCV7 period in the 6–60 years age group. In the older adult group, non-susceptibility to COT, CLI, CFT and CFX was significantly reduced in the EPI-PCV13 compared to the Private-PCV7 period (Fig. S2D).

**Fig. 6.**
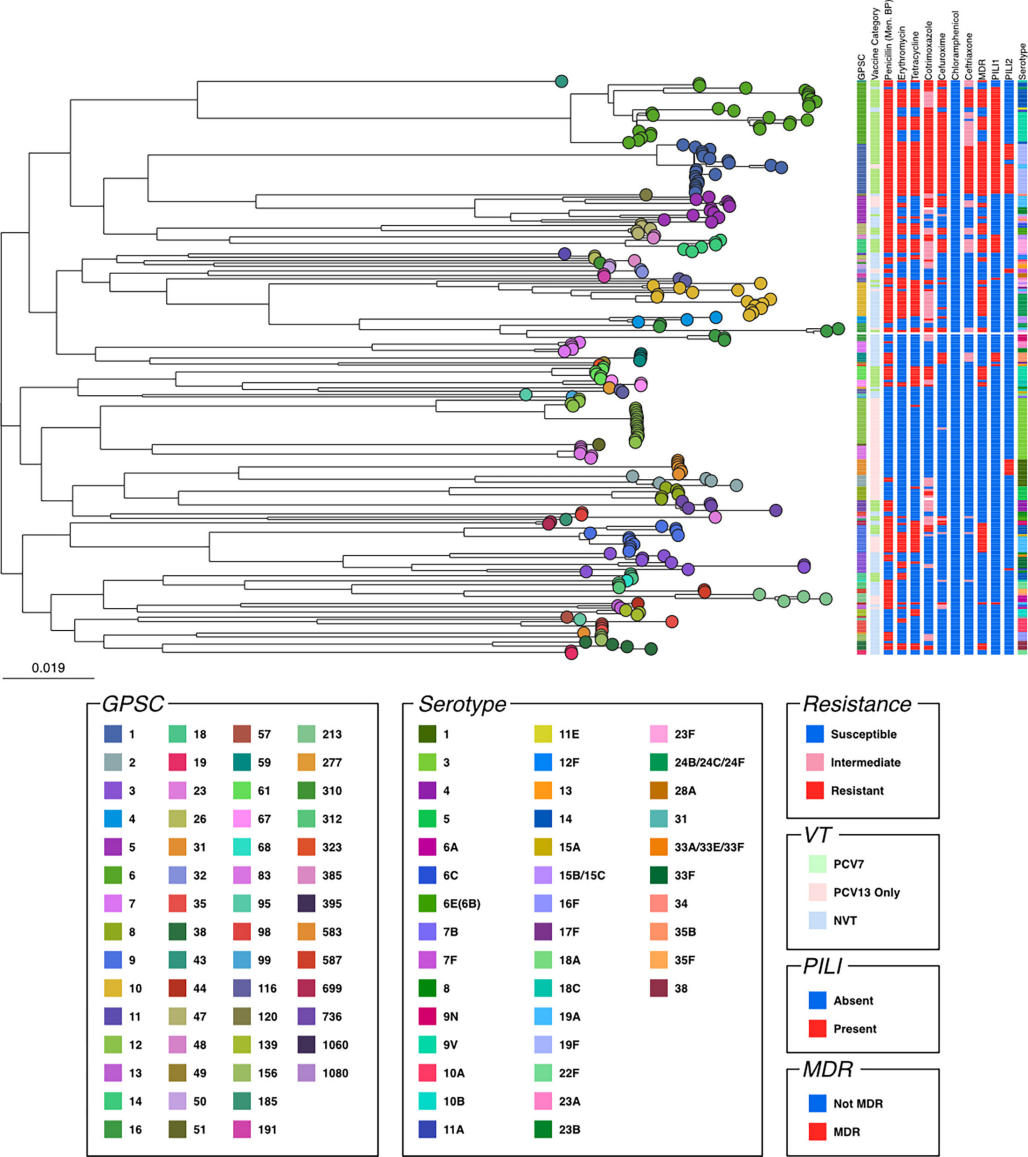
Maximum-likelihood tree of *S. pneumoniae* isolates collected from IPD patients (*n*=273) in Lebanon, 2003–2025. The tree nodes are coloured by GPSC. Interactive figures can be visualized at https://microreact.org/project/lebanon-gps. Abbreviations: AMO, amoxicillin; AMR, antimicrobial resistance; CFX, cefuroxime; CHL, chloramphenicol; CLI, clindamycin; COT, cotrimoxazole; ERY, erythromycin; GPSC, global pneumococcal sequence clusters; MDR, multidrug resistance; NVT, non-vaccine type; PEN, penicillin; PCV, pneumococcal conjugate vaccines; PILI1, Type 1 pili; PILI2, Type 2 pili; TET, tetracycline. PCV7 refers to serotypes covered by the 7-valent PCV (PCV7); PCV13 represents additional serotypes covered by the 13-valent PCV (PCV13), but not PCV7; NVTs are serotypes that are not covered by PCV13.

A total of 69 out of 273 (25.2%) isolates were simultaneously susceptible to PEN, ERY, TET, COT and CHL. They were PCV13-only serotypes (*n*=39/69; 56.5%), followed by NVT (*n*=29/69; 42.0%) and only one isolate (1.4%) categorized as PCV7 (Fig. 6). All GPSC1 (*n*=21; 100%), GPSC9 (*n*=15; 100%), GPSC61 (*n*=6; 100%), GPSC14 (*n*=5; 100%) and the majority of GPSC10 isolates (*n*=13/14; 92.8%) were MDR. PILI1 (Type 1 pili) and PILI2 (Type 2 pili) were co-detected in all GPSC1 genomes (*n*=21/21; 100.0%), in which the majority were ST320 (*n*=18/21; 85.7%). More than half of the *S. pneumoniae* isolates that were positive for PILI1 but negative for PILI2 were MDR (*n*=22/39; 56.4%). None of the *S. pneumoniae* isolates in which only PILI2 was detected were MDR.

## Discussion

The recommendations on PCV vaccination for children in Lebanon have changed frequently over the past two decades. Concerningly, data from this study suggest that the recent policy change from PCV13 to PCV10 (Pneumosil) in the Lebanese EPI may lead to an increase in IPD. We show that the projected serotype coverage of PCV10 (Pneumosil) is approximately half that of PCV13 in the EPI-PCV13 period, which represents the most recent period in this study. Throughout the study period, serotype 3 was the most prevalent IPD serotype, a trend consistent with reports from the USA [24], Scotland [25], Canada [26] and the Middle East [27], including Qatar [28]. Serotype 3 persistence despite PCV13 implementation was reported in Europe [29,30] and Asia [31]. Similarly, we have identified serotype 3 as the predominant serotype during the EPI-PCV13 period in Lebanon. Furthermore, our analysis identified a significant increase in NVT prevalence after PCV7 and PCV13 introduction, which is consistent with local [14] and global reports [32,33].

Vaccinating vulnerable older adult populations with PCV and/or pneumococcal polysaccharide vaccine in Lebanon emerges as a priority. In this population, PCV7 serotypes were associated with significantly higher mortality compared to NVTs. A study by Grau *et al.* also reported an association between IPD caused by PCV7 serotypes and death [34]. Moreover, PCV7 serotypes were identified as risk factors for mortality among IPD patients aged between 5 and 64 years in Europe [35]. Interestingly, although PCV7 serotypes accounted for only 33% of IPD cases among older adult patients included in this study, we observed the highest IPD-related mortality in the older adult group. Backhaus *et al.* similarly showed that over a 45-year period in Sweden, IPD-specific mortality tripled among individuals aged ≥65 years [36]. A complementary older adult vaccination programme alongside the childhood vaccination programme may be an effective option [37].

While most studies from Middle East and North Africa countries have focused on serotype distribution in IPD, only one has characterized the underlying genomic lineages [38]. The most predominant GPSC in this study was GPSC6, GPSC1, GPSC12 and GPSC10. The majority of GPSC12 genomes were detected during the EPI-PCV13 period (*n*=20/25; 80%), consistent with findings from Norway, where GPSC12 also expanded following PCV introduction [39]. Globally, GPSC1 and GPSC6 are among the most widespread pneumococcal lineages [20], with GPSC1 being a common MDR lineage [10,11, 40]. Moreover, GPSC1 was the only lineage in which PILI1 and PILI2 were co-detected. The presence of both pili may enhance bacterial adhesion and persistence in the host [41], potentially increasing the duration of antibiotic exposure and promoting the development of multidrug resistance. Although in the Private-PCV7 period, all GPSC10 genomes expressed VTs, the majority of GPSC10 in the Private-PCV13 and EPI-PCV13 periods expressed serogroup 24. In the EPI-PCV13 period, serogroup 24 was only expressed by GPSC10. Serotype 24F isolates previously reported in Lebanon have been characterized as highly virulent and MDR [42], and the emergence of GPSC10 expressing serotype 24F following PCV13 introduction as an invasive MDR lineage was also previously reported elsewhere [43].

In Lebanon, the treatment of choice for *S. pneumoniae* is AMO or third-generation cephalosporins. However, over the study periods, we found that the prevalence of resistance to antibiotics such as PEN, ERY, CHL, TET and MDR significantly decreased. Whilst encouraging to see, the reasons for this are complex; the availability of vaccinations may have led to fewer pneumococcal infections, and so fewer antibiotics being prescribed to treat these infections and a reduction in the selective pressure the bacteria are under. Additionally, a decrease in lineages with many MDR isolates, such as GPSC1, may have contributed to this decrease. Similar to our findings, a global meta-regression analysis demonstrated significant reductions in resistance of *S. pneumoniae* to PEN, COT and third-generation cephalosporins, over a 10-year period following the implementation of any PCV [44]. However, changes in the availability of antimicrobials and antibiotic prescription guidelines may also have had an impact. Despite the reduction in AMR after PCV use, more than half of the isolates from the EPI-PCV13 period were still resistant to PEN (55.9%) and COT (50.3%). This finding underscores the importance of continued antimicrobial stewardship efforts alongside sustained vaccine coverage.

One of the strong points of this study is its multicentre design, with samples collected from five regions across Lebanon. Moreover, focusing specifically on invasive disease cases ensured consistency in clinical presentation and relevance to public health impact. Another notable strength is the extended duration of surveillance, spanning over two decades (2003–2025), and providing unique insights into the temporal trends of pneumococcal disease.

While our study provides valuable insights, it has several limitations. The sample size became relatively small when performing subgroup analyses. Hence, we could not meaningfully quantify the change in individual serotypes or GPSC over the different vaccine periods due to the lack of statistical power. Moreover, not all available isolates were sequenced, as many failed to grow in culture, did not yield sufficient DNA upon extraction or were affected by contamination, which may have limited the breadth of genomic diversity captured. Sequencing was not re-attempted for these samples due to resource constraints. Furthermore, data on individual vaccination status were unavailable, restricting the ability to assess vaccine effectiveness at the patient level. Finally, the intensity of *S. pneumoniae* surveillance increased over the study period, as additional hospitals were progressively integrated into the LIPSP network, introducing temporal biases. However, events such as the wars in 2006 and 2024, along with the economic crisis that began in late 2019, likely contributed to reduced hospital admissions for infectious diseases. As of 2025, Lebanon is still dealing with the economic fallout. These factors may have negatively impacted IPD surveillance during the affected periods. Furthermore, prescribing practices evolved over time, with a decrease in the use of COT. Notably, antibiotics remain widely accessible without prescription in Lebanon, particularly in outpatient clinics and rural areas. A substantial proportion of samples originated from hospitals in Beirut, attributed to a tendency for patients, including those living outside Beirut, to seek care in the capital’s hospitals.

## Conclusion

Over the 22-year study period, we observed a significant decline in PCV7 serotypes and AMR, while the prevalence of NVTs increased. Our findings highlight that PCV10 (Pneumosil) offers considerably reduced serotype coverage than PCV13 in the most recent study period, raising concern as the EPI shifts back to PCV10 (Pneumosil). Given the high mortality rate detected in older adult IPD patients, including this population in the immunization programme may be beneficial. Genomic surveillance will continue through LIPSP in the coming years to track the serotype distribution, lineages and AMR patterns of invasive *S. pneumoniae* bacteria in Lebanon.

## Supplementary material

10.1099/mgen.0.001664 Uncited Supplementary Material 1.

## Abbreviations

AMO: amoxicillin
AMR: antimicrobial resistance
CFT: ceftriaxone
CFX: cefuroxime
CHL: chloramphenicol
CIDR: Center for Infectious Diseases Research
CLI: clindamycin
CLSI: Clinical Laboratory Standards Institute
COT: cotrimoxazole
EPI: Expanded Program On Immunization
ERY: erythromycin
FQ: fluoroquinolones
GPS: global pneumococcal sequencing
GPSCs: GPS clusters
IPD: invasive pneumococcal diseases
IRB: Institutional Review Board
LIPSP: Lebanese Inter-Hospital Pneumococcal Surveillance Program
MDR: multidrug resistance
NVT: non-vaccine types
ONT: Oxford Nanopore Technologies
PBP: PEN-binding protein
PCV: pneumococcal conjugate vaccines
PCV7: 7-valent PCV
PCV13: 13-valent PCV
PEN: penicillin
PILI1: Type 1 pili
PILI2: Type 2 pili
QC: quality control
STs: sequence types
TET: tetracycline
VT: vaccine type
WGS: whole-genome sequencing
