# Supplementary material for: Genomic characterization of invasive disease-causing Streptococcus pneumoniae in Lebanon, 2003–2025
Source: Microb Genom. 2026 Mar 13;12(3):001664. doi: 10.1099/mgen.0.001664 (PMC12987499; doi:10.1099/mgen.0.001664)
Supplement: Uncited Supplementary Material 1. [file mgen-12-01664-s001.pdf]

Genomic Characterization of Invasive Disease-Causing *Streptococcus pneumoniae* in Lebanon 2003-2025

Supplementary Figures and Tables

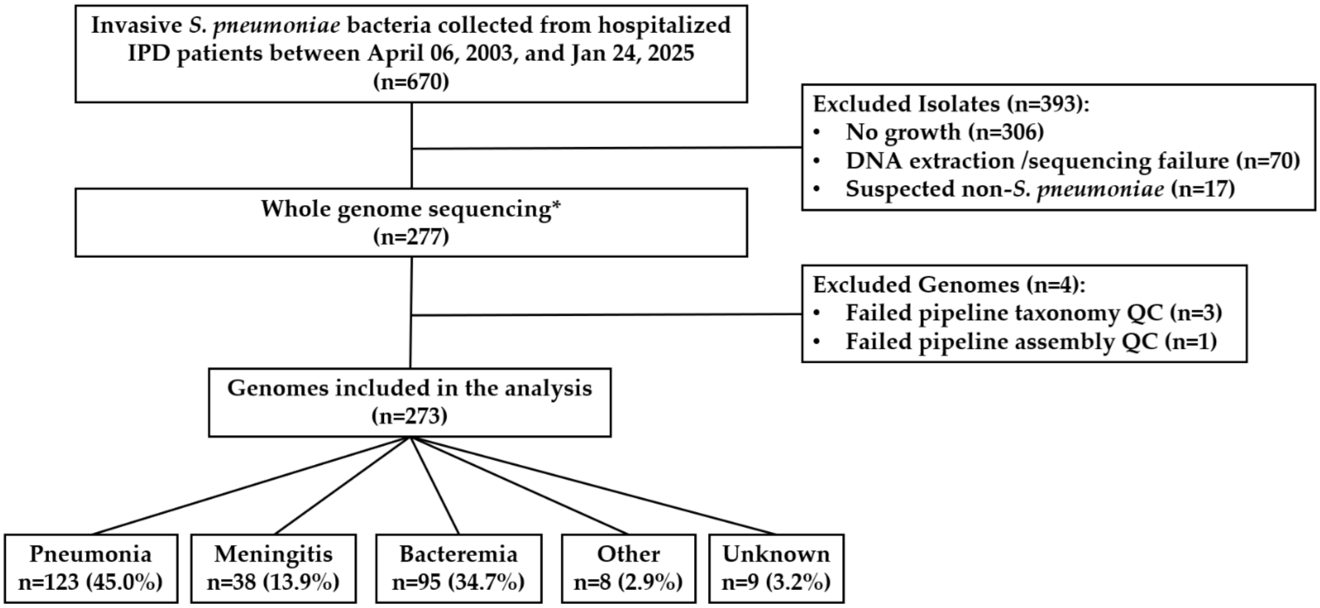

Figure S1: Flowchart of the study design

* successfully sequenced and generated reliable data that entered the analysis pipeline.

Table S1: AMR and MDR prevalence according to PCV7, PCV13-only and NVTs.

	PCV7 (n=91)	PCV13-only (n=76)	NVTs (n=106)	Total (n=273)	p-value
**Penicillin**	81 (89.0%)	27 (35.5%)	62 (58.4%)	170 (62.2%)	**<0.0001**
**Cefuroxime**	62 (68.1%)	11 (14.4%)	12 (11.3%)	85 (31.1%)	**<0.0001**
**Ceftriaxone**	59 (64.8%)	6 (7.8%)	3 (2.8%)	68 (24.9%)	**<0.0001**
**Clindamycin**	41 (45.0%)	11 (14.4%)	20 (18.8%)	72 (26.3%)	**<0.0001**
**Erythromycin**	61 (67.0%)	15 (19.7%)	29 (27.3%)	105 (38.4%)	**<0.0001**
**Tetracycline**	65 (71.4%)	15 (19.7%)	29 (27.3%)	109 (39.9%)	**<0.0001**
**Cotrimoxazole**	79 (86.8%)	17/75 (22.6%)	46/103 (44.6%)	142/269 (52.7%)	**<0.0001**
**Chloramphenicol**	1 (1.0%)	1 (1.3%)	1 (0.9%)	3 (1.0%)	**NS**
**Fluoroquinolone**	1 (1.0%)	0 (0.0%)	0 (0.0%)	1 (0.3%)	**NS**
**MDR**	62 (68.1%)	15 (19.7%)	23 (21.60%)	100 (36.6%)	**<0.0001**

Table S2: Trends in antibiotic non-susceptibility among NVT (n=105) over the Private-PCV13 and EPI-PCV13 vaccine periods.

	Private-PCV13 (n=27)	EPI-PCV13 (n=78)	Total* (n=105)	p-value
**Penicillin**	14 (51.8%)	47 (60.2%)	61 (58.0%)	NS
**Cefuroxime**	3 (11.11%)	9 (11.5%)	12 (11.4%)	NS
**Ceftriaxone**	2 (7.4%)	1 (1.2%)	3 (2.8%)	NS
**Clindamycin**	4 (14.8%)	16 (20.5%)	20 (19.0%)	NS
**Erythromycin**	4 (14.8%)	25 (32.0%)	29 (27.6%)	NS
**Tetracycline**	6 (22.22%)	23 (29.4%)	29 (27.6%)	NS
**Cotrimoxazole**	7/26 (26.9%)	38/76 (50.0%)	45/102 (44.11%)	0.0408
**Chloramphenicol**	1 (3.7%)	0 (0%)	1 (0.95%)	NS
**Fluoroquinolone**	0 (0%)	0 (0%)	0 (0%)	NS
**MDR**	4 (14.8%)	19 (24.3%)	23 (21.9%)	NS

*Only one NVT genome was available in the Private-PCV7 period and excluded from the table.

Significant differences were calculated using the Chi Square or Fisher's exact test between the Private-PCV13 and EPI-PCV13 periods. Non-significant values are indicated with NS.

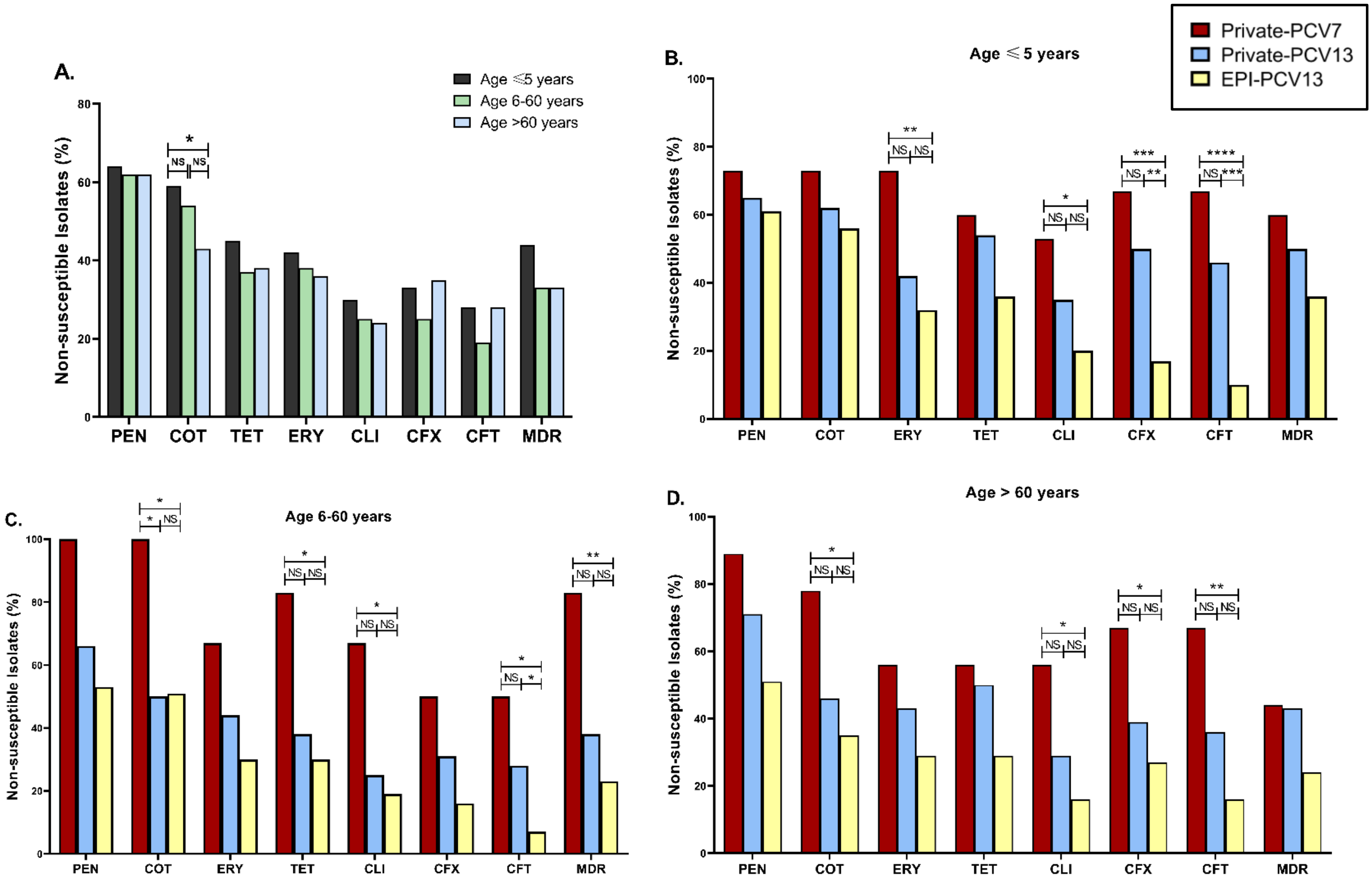

Figure S2: Antimicrobial resistance among *S. pneumoniae* isolates stratified by age groups (A). The rates of MDR and AMR among the ≤ 5 years (B), 6-60 years (C) and > 60 years (D) age groups over the three study periods.

Significant differences were calculated using Chi Squared or Fisher's exact test. Bars without a label showed no significant differences (p> 0.05) between the age groups.

Significant values are indicated with * (p< 0.05) or ** (p< 0.01) or ***(p< 0.001). Non-significant values are indicated with NS. In this figure, all non-susceptible isolates, including those with intermediate and resistant genotypes, were classified as resistant.

In the ≤ 5 years age group, there were 15 isolates from the Private-PCV7 period, 26 from the Private-PCV13 period and 59 from the EPI-PCV13 period. In the 6-60 years age group, there were 6 isolates from the Private-PCV7 period, 32 from the Private-PCV13 period and 43 from the EPI-PCV13 period. In the > 60 years age group, there were 9 isolates from the Private-PCV7 period, 28 from the Private-PCV13 period and 49 from the EPI-PCV13 period.

Abbreviations: CFT, Ceftriaxone; CFX, Cefuroxime; CLI: Clindamycin; COT, cotrimoxazole; ERY: Erythromycin; MDR, Multidrug resistant; PEN: Penicillin; TET: Tetracycline

Table S3: ENA Accession IDs of genomes sequenced locally at the CIDR lab

run_accession	sample_accession	experiment_accession	study_accession	tax_id	scientific_name
ERR15171075	SAMEA118586224	ERX14576527	PRJEB88880	1313	*Streptococcus pneumoniae*
ERR15171091	SAMEA118586227	ERX14576543	PRJEB88880	1313	*Streptococcus pneumoniae*
ERR15171121	SAMEA118586202	ERX14576573	PRJEB88880	1313	*Streptococcus pneumoniae*
ERR15171125	SAMEA118586204	ERX14576577	PRJEB88880	1313	*Streptococcus pneumoniae*
ERR15171128	SAMEA118586234	ERX14576580	PRJEB88880	1313	*Streptococcus pneumoniae*
ERR15171137	SAMEA118586242	ERX14576589	PRJEB88880	1313	*Streptococcus pneumoniae*
ERR15171138	SAMEA118586206	ERX14576590	PRJEB88880	1313	*Streptococcus pneumoniae*
ERR15171139	SAMEA118586207	ERX14576591	PRJEB88880	1313	*Streptococcus pneumoniae*
ERR15171141	SAMEA118586209	ERX14576593	PRJEB88880	1313	*Streptococcus pneumoniae*
ERR15171142	SAMEA118586210	ERX14576594	PRJEB88880	1313	*Streptococcus pneumoniae*
ERR15171146	SAMEA118586214	ERX14576598	PRJEB88880	1313	*Streptococcus pneumoniae*
ERR15171147	SAMEA118586215	ERX14576599	PRJEB88880	1313	*Streptococcus pneumoniae*
ERR15171153	SAMEA118586220	ERX14576605	PRJEB88880	1313	*Streptococcus pneumoniae*
ERR15314068	SAMEA118586187	ERX14720396	PRJEB88880	1313	*Streptococcus pneumoniae*
ERR15314069	SAMEA118586188	ERX14720397	PRJEB88880	1313	*Streptococcus pneumoniae*
ERR15171069	SAMEA118586221	ERX14576521	PRJEB88880	1313	*Streptococcus pneumoniae*
ERR15171070	SAMEA118586195	ERX14576522	PRJEB88880	1313	*Streptococcus pneumoniae*
ERR15171090	SAMEA118586226	ERX14576542	PRJEB88880	1313	*Streptococcus pneumoniae*
ERR15171117	SAMEA118586199	ERX14576569	PRJEB88880	1313	*Streptococcus pneumoniae*
ERR15171118	SAMEA118586229	ERX14576570	PRJEB88880	1313	*Streptococcus pneumoniae*
ERR15171124	SAMEA118586231	ERX14576576	PRJEB88880	1313	*Streptococcus pneumoniae*
ERR15171127	SAMEA118586233	ERX14576579	PRJEB88880	1313	*Streptococcus pneumoniae*
ERR15171132	SAMEA118586205	ERX14576584	PRJEB88880	1313	*Streptococcus pneumoniae*
ERR15171133	SAMEA118586238	ERX14576585	PRJEB88880	1313	*Streptococcus pneumoniae*
ERR15171134	SAMEA118586239	ERX14576586	PRJEB88880	1313	*Streptococcus pneumoniae*
ERR15171144	SAMEA118586212	ERX14576596	PRJEB88880	1313	*Streptococcus pneumoniae*
ERR15171151	SAMEA118586219	ERX14576603	PRJEB88880	1313	*Streptococcus pneumoniae*
ERR15314067	SAMEA118586186	ERX14720395	PRJEB88880	1313	*Streptococcus pneumoniae*
ERR15314070	SAMEA118586189	ERX14720398	PRJEB88880	1313	*Streptococcus pneumoniae*
ERR15314071	SAMEA118586190	ERX14720399	PRJEB88880	1313	*Streptococcus pneumoniae*
ERR15314073	SAMEA118586192	ERX14720401	PRJEB88880	1313	*Streptococcus pneumoniae*
ERR15314075	SAMEA118586194	ERX14720403	PRJEB88880	1313	*Streptococcus pneumoniae*
ERR15171072	SAMEA118586196	ERX14576524	PRJEB88880	1313	*Streptococcus pneumoniae*
ERR15171073	SAMEA118586197	ERX14576525	PRJEB88880	1313	*Streptococcus pneumoniae*
ERR15171074	SAMEA118586223	ERX14576526	PRJEB88880	1313	*Streptococcus pneumoniae*
ERR15171088	SAMEA118586225	ERX14576540	PRJEB88880	1313	*Streptococcus pneumoniae*
ERR15171116	SAMEA118586228	ERX14576568	PRJEB88880	1313	*Streptococcus pneumoniae*

ERR15171119	SAMEA118586200	ERX14576571	PRJEB88880	1313	*Streptococcus pneumoniae*
ERR15171120	SAMEA118586201	ERX14576572	PRJEB88880	1313	*Streptococcus pneumoniae*
ERR15171122	SAMEA118586230	ERX14576574	PRJEB88880	1313	*Streptococcus pneumoniae*
ERR15171123	SAMEA118586203	ERX14576575	PRJEB88880	1313	*Streptococcus pneumoniae*
ERR15171126	SAMEA118586232	ERX14576578	PRJEB88880	1313	*Streptococcus pneumoniae*
ERR15171129	SAMEA118586235	ERX14576581	PRJEB88880	1313	*Streptococcus pneumoniae*
ERR15171130	SAMEA118586236	ERX14576582	PRJEB88880	1313	*Streptococcus pneumoniae*
ERR15171131	SAMEA118586237	ERX14576583	PRJEB88880	1313	*Streptococcus pneumoniae*
ERR15171135	SAMEA118586240	ERX14576587	PRJEB88880	1313	*Streptococcus pneumoniae*
ERR15171136	SAMEA118586241	ERX14576588	PRJEB88880	1313	*Streptococcus pneumoniae*
ERR15171140	SAMEA118586208	ERX14576592	PRJEB88880	1313	*Streptococcus pneumoniae*
ERR15171143	SAMEA118586211	ERX14576595	PRJEB88880	1313	*Streptococcus pneumoniae*
ERR15171145	SAMEA118586213	ERX14576597	PRJEB88880	1313	*Streptococcus pneumoniae*
ERR15171148	SAMEA118586216	ERX14576600	PRJEB88880	1313	*Streptococcus pneumoniae*
ERR15171149	SAMEA118586217	ERX14576601	PRJEB88880	1313	*Streptococcus pneumoniae*
ERR15171150	SAMEA118586218	ERX14576602	PRJEB88880	1313	*Streptococcus pneumoniae*
ERR15314065	SAMEA118586184	ERX14720393	PRJEB88880	1313	*Streptococcus pneumoniae*
ERR15314066	SAMEA118586185	ERX14720394	PRJEB88880	1313	*Streptococcus pneumoniae*
ERR15314072	SAMEA118586191	ERX14720400	PRJEB88880	1313	*Streptococcus pneumoniae*
ERR15314074	SAMEA118586193	ERX14720402	PRJEB88880	1313	*Streptococcus pneumoniae*
ERR15171071	SAMEA118586222	ERX14576523	PRJEB88880	1313	*Streptococcus pneumoniae*
ERR15171076	SAMEA118586198	ERX14576528	PRJEB88880	1313	*Streptococcus pneumoniae*

Table S4: ENA Accession IDs of genomes sequenced at the Wellcome Sanger Institute

ebi_run_accession	sample_accession_number	ebi_submission_accession	study_accession_number	*sample_common_name*
ERR11280133	ERS14831647	ERA23129176	ERP001505	*Streptococcus pneumoniae*
ERR14365600	ERS22549823	ERA31142933	ERP001505	*Streptococcus pneumoniae*
ERR11280479	ERS14831636	ERA23129176	ERP001505	*Streptococcus pneumoniae*
ERR11280158	ERS14831801	ERA23129176	ERP001505	*Streptococcus pneumoniae*
ERR11280172	ERS14831808	ERA23129176	ERP001505	*Streptococcus pneumoniae*
ERR11280126	ERS14831793	ERA23129176	ERP001505	*Streptococcus pneumoniae*
ERR11280429	ERS14831755	ERA23129176	ERP001505	*Streptococcus pneumoniae*
ERR11010295	ERS14651704	ERA20775729	ERP001505	*Streptococcus_pneumoniae*
ERR11280198	ERS14831814	ERA23129176	ERP001505	*Streptococcus pneumoniae*
ERR14365555	ERS22549774	ERA31142933	ERP001505	*Streptococcus pneumoniae*
ERR11280299	ERS14831703	ERA23129176	ERP001505	*Streptococcus pneumoniae*
ERR11280230	ERS14831823	ERA23129176	ERP001505	*Streptococcus pneumoniae*
ERR12768034	ERS17991853	ERA29530219	ERP001505	*Streptococcus pneumoniae*
ERR11280163	ERS14831658	ERA23129176	ERP001505	*Streptococcus pneumoniae*
ERR12768035	ERS17991851	ERA29530219	ERP001505	*Streptococcus pneumoniae*

ERR11010217	ERS14651702	ERA20775729	ERP001505	*Streptococcus_pneumoniae*
ERR12768111	ERS17991926	ERA29530219	ERP001505	*Streptococcus pneumoniae*
ERR11280389	ERS14831734	ERA23129176	ERP001505	*Streptococcus pneumoniae*
ERR14365551	ERS22549780	ERA31142933	ERP001505	*Streptococcus pneumoniae*
ERR11280131	ERS14831646	ERA23129176	ERP001505	*Streptococcus pneumoniae*
ERR12768091	ERS17991889	ERA29530219	ERP001505	*Streptococcus pneumoniae*
ERR12768108	ERS17991923	ERA29530219	ERP001505	*Streptococcus pneumoniae*
ERR12768110	ERS17991925	ERA29530219	ERP001505	*Streptococcus pneumoniae*
ERR11280323	ERS14831711	ERA23129176	ERP001505	*Streptococcus pneumoniae*
ERR11582627	ERS15530219	ERA24269131	ERP001505	*Streptococcus pneumoniae*
ERR11280255	ERS14831688	ERA23129176	ERP001505	*Streptococcus pneumoniae*
ERR11280489	ERS14831642	ERA23129176	ERP001505	*Streptococcus pneumoniae*
ERR12908413	ERS17742558	ERA29605220	ERP001505	*Streptococcus pneumoniae*
ERR12768085	ERS17991877	ERA29530219	ERP001505	*Streptococcus pneumoniae*
ERR14365622	ERS22549715	ERA31142933	ERP001505	*Streptococcus pneumoniae*
ERR11280423	ERS14831748	ERA23129176	ERP001505	*Streptococcus pneumoniae*
ERR13358795	ERS19862770	ERA30698859	ERP001505	*Streptococcus pneumoniae*
ERR14365587	ERS22549590	ERA31142933	ERP001505	*Streptococcus pneumoniae*
ERR11280451	ERS14831760	ERA23129176	ERP001505	*Streptococcus pneumoniae*
ERR11280487	ERS14831641	ERA23129176	ERP001505	*Streptococcus pneumoniae*
ERR11280129	ERS14831644	ERA23129176	ERP001505	*Streptococcus pneumoniae*
ERR11280459	ERS14831764	ERA23129176	ERP001505	*Streptococcus pneumoniae*
ERR11280334	ERS14831720	ERA23129176	ERP001505	*Streptococcus pneumoniae*
ERR11280164	ERS14831804	ERA23129176	ERP001505	*Streptococcus pneumoniae*
ERR11280203	ERS14831676	ERA23129176	ERP001505	*Streptococcus pneumoniae*
ERR12768082	ERS17991873	ERA29530219	ERP001505	*Streptococcus pneumoniae*
ERR11280162	ERS14831803	ERA23129176	ERP001505	*Streptococcus pneumoniae*
ERR11280477	ERS14831635	ERA23129176	ERP001505	*Streptococcus pneumoniae*
ERR11280346	ERS14831746	ERA23129176	ERP001505	*Streptococcus pneumoniae*
ERR11280353	ERS14831722	ERA23129176	ERP001505	*Streptococcus pneumoniae*
ERR12768038	ERS17991865	ERA29530219	ERP001505	*Streptococcus pneumoniae*
ERR11280421	ERS14831747	ERA23129176	ERP001505	*Streptococcus pneumoniae*
ERR12768046	ERS17991904	ERA29530219	ERP001505	*Streptococcus pneumoniae*
ERR12768076	ERS17991867	ERA29530219	ERP001505	*Streptococcus pneumoniae*
ERR11280159	ERS14831655	ERA23129176	ERP001505	*Streptococcus pneumoniae*
ERR11280161	ERS14831657	ERA23129176	ERP001505	*Streptococcus pneumoniae*
ERR12768054	ERS17991916	ERA29530219	ERP001505	*Streptococcus pneumoniae*
ERR11280184	ERS14831820	ERA23129176	ERP001505	*Streptococcus pneumoniae*
ERR14365583	ERS22549764	ERA31142933	ERP001505	*Streptococcus pneumoniae*
ERR11582628	ERS15530220	ERA24269131	ERP001505	*Streptococcus pneumoniae*

ERR14365678	ERS22549732	ERA31142933	ERP001505	*Streptococcus pneumoniae*
ERR11280361	ERS14831727	ERA23129176	ERP001505	*Streptococcus pneumoniae*
ERR11280232	ERS14831824	ERA23129176	ERP001505	*Streptococcus pneumoniae*
ERR11280284	ERS14831693	ERA23129176	ERP001505	*Streptococcus pneumoniae*
ERR11280168	ERS14831805	ERA23129176	ERP001505	*Streptococcus pneumoniae*
ERR11280176	ERS14831788	ERA23129176	ERP001505	*Streptococcus pneumoniae*
ERR11010219	ERS14651700	ERA20775729	ERP001505	*Streptococcus_pneumoniae*
ERR12908415	ERS17742560	ERA29605220	ERP001505	*Streptococcus pneumoniae*
ERR11280447	ERS14831757	ERA23129176	ERP001505	*Streptococcus pneumoniae*
ERR11010263	ERS14651729	ERA20775729	ERP001505	*Streptococcus_pneumoniae*
ERR12908396	ERS17742557	ERA29605220	ERP001505	*Streptococcus pneumoniae*
ERR12768080	ERS17991872	ERA29530219	ERP001505	*Streptococcus pneumoniae*
ERR11280336	ERS14831724	ERA23129176	ERP001505	*Streptococcus pneumoniae*
ERR12768105	ERS17991911	ERA29530219	ERP001505	*Streptococcus pneumoniae*
ERR11280370	ERS14831754	ERA23129176	ERP001505	*Streptococcus pneumoniae*
ERR14365652	ERS22549725	ERA31142933	ERP001505	*Streptococcus pneumoniae*
ERR11280169	ERS14831663	ERA23129176	ERP001505	*Streptococcus pneumoniae*
ERR11280138	ERS14831800	ERA23129176	ERP001505	*Streptococcus pneumoniae*
ERR11280222	ERS14831819	ERA23129176	ERP001505	*Streptococcus pneumoniae*
ERR13358793	ERS19862767	ERA30698859	ERP001505	*Streptococcus pneumoniae*
ERR12768092	ERS17991888	ERA29530219	ERP001505	*Streptococcus pneumoniae*
ERR11280165	ERS14831659	ERA23129176	ERP001505	*Streptococcus pneumoniae*
ERR11280383	ERS14831733	ERA23129176	ERP001505	*Streptococcus pneumoniae*
ERR11010225	ERS14651705	ERA20775729	ERP001505	*Streptococcus_pneumoniae*
ERR11280366	ERS14831751	ERA23129176	ERP001505	*Streptococcus pneumoniae*
ERR11280130	ERS14831795	ERA23129176	ERP001505	*Streptococcus pneumoniae*
ERR11280139	ERS14831653	ERA23129176	ERP001505	*Streptococcus pneumoniae*
ERR14365651	ERS22549617	ERA31142933	ERP001505	*Streptococcus pneumoniae*
ERR14365617	ERS22549601	ERA31142933	ERP001505	*Streptococcus pneumoniae*
ERR11280204	ERS14831818	ERA23129176	ERP001505	*Streptococcus pneumoniae*
ERR12768065	ERS17991850	ERA29530219	ERP001505	*Streptococcus pneumoniae*
ERR12768051	ERS17991912	ERA29530219	ERP001505	*Streptococcus pneumoniae*
ERR11280363	ERS14831728	ERA23129176	ERP001505	*Streptococcus pneumoniae*
ERR12768059	ERS17991927	ERA29530219	ERP001505	*Streptococcus pneumoniae*
ERR14365836	ERS22549834	ERA31142933	ERP001505	*Streptococcus pneumoniae*
ERR11280196	ERS14831812	ERA23129176	ERP001505	*Streptococcus pneumoniae*
ERR11280456	ERS14831631	ERA23129176	ERP001505	*Streptococcus pneumoniae*
ERR14365834	ERS22549833	ERA31142933	ERP001505	*Streptococcus pneumoniae*
ERR11280182	ERS14831811	ERA23129176	ERP001505	*Streptococcus pneumoniae*
ERR11280483	ERS14831640	ERA23129176	ERP001505	*Streptococcus pneumoniae*

ERR12768112	ERS17991928	ERA29530219	ERP001505	Streptococcus pneumoniae
ERR11280194	ERS14831813	ERA23129176	ERP001505	Streptococcus pneumoniae
ERR11010257	ERS14651728	ERA20775729	ERP001505	Streptococcus_pneumoniae
ERR11280486	ERS14831774	ERA23129176	ERP001505	Streptococcus pneumoniae
ERR14365655	ERS22549619	ERA31142933	ERP001505	Streptococcus pneumoniae
ERR11280244	ERS14831648	ERA23129176	ERP001505	Streptococcus pneumoniae
ERR12768101	ERS17991901	ERA29530219	ERP001505	Streptococcus pneumoniae
ERR12768057	ERS17991920	ERA29530219	ERP001505	Streptococcus pneumoniae
ERR14365572	ERS22549813	ERA31142933	ERP001505	Streptococcus pneumoniae
ERR11280166	ERS14831806	ERA23129176	ERP001505	Streptococcus pneumoniae
ERR11280171	ERS14831666	ERA23129176	ERP001505	Streptococcus pneumoniae
ERR12768078	ERS17991869	ERA29530219	ERP001505	Streptococcus pneumoniae
ERR11280316	ERS14831717	ERA23129176	ERP001505	Streptococcus pneumoniae
ERR14365807	ERS22549675	ERA31142933	ERP001505	Streptococcus pneumoniae
ERR11010251	ERS14651722	ERA20775729	ERP001505	Streptococcus_pneumoniae
ERR11280180	ERS14831789	ERA23129176	ERP001505	Streptococcus pneumoniae
ERR11280242	ERS14831649	ERA23129176	ERP001505	Streptococcus pneumoniae
ERR11280227	ERS14831683	ERA23129176	ERP001505	Streptococcus pneumoniae
ERR12768053	ERS17991913	ERA29530219	ERP001505	Streptococcus pneumoniae
ERR12768042	ERS17991886	ERA29530219	ERP001505	Streptococcus pneumoniae
ERR11009894	ERS14310441	ERA20775729	ERP001505	Streptococcus pneumoniae
ERR11010317	ERS14651721	ERA20775729	ERP001505	Streptococcus_pneumoniae
ERR11280173	ERS14831665	ERA23129176	ERP001505	Streptococcus pneumoniae
ERR11280205	ERS14831677	ERA23129176	ERP001505	Streptococcus pneumoniae
ERR11009896	ERS14310445	ERA20775729	ERP001505	Streptococcus pneumoniae
ERR11009890	ERS14310439	ERA20775729	ERP001505	Streptococcus pneumoniae
ERR11280167	ERS14831662	ERA23129176	ERP001505	Streptococcus pneumoniae
ERR11280178	ERS14831790	ERA23129176	ERP001505	Streptococcus pneumoniae
ERR11010191	ERS14651698	ERA20775729	ERP001505	Streptococcus_pneumoniae
ERR12768104	ERS17991909	ERA29530219	ERP001505	Streptococcus pneumoniae
ERR12768058	ERS17991922	ERA29530219	ERP001505	Streptococcus pneumoniae
ERR12768083	ERS17991875	ERA29530219	ERP001505	Streptococcus pneumoniae
ERR12908393	ERS17742554	ERA29605220	ERP001505	Streptococcus pneumoniae
ERR12768074	ERS17991864	ERA29530219	ERP001505	Streptococcus pneumoniae
ERR11280291	ERS14831704	ERA23129176	ERP001505	Streptococcus pneumoniae
ERR14365638	ERS22549769	ERA31142933	ERP001505	Streptococcus pneumoniae
ERR11280460	ERS14831633	ERA23129176	ERP001505	Streptococcus pneumoniae
ERR14365907	ERS22549753	ERA31142933	ERP001505	Streptococcus pneumoniae
ERR11280331	ERS14831721	ERA23129176	ERP001505	Streptococcus pneumoniae
ERR11280135	ERS14831651	ERA23129176	ERP001505	Streptococcus pneumoniae

ERR11582626	ERS15530216	ERA24269131	ERP001505	*Streptococcus pneumoniae*
ERR12768086	ERS17991880	ERA29530219	ERP001505	*Streptococcus pneumoniae*
ERR12768040	ERS17991879	ERA29530219	ERP001505	*Streptococcus pneumoniae*
ERR11280481	ERS14831638	ERA23129176	ERP001505	*Streptococcus pneumoniae*
ERR12768079	ERS17991871	ERA29530219	ERP001505	*Streptococcus pneumoniae*
ERR12768081	ERS17991874	ERA29530219	ERP001505	*Streptococcus pneumoniae*
ERR12768050	ERS17991910	ERA29530219	ERP001505	*Streptococcus pneumoniae*
ERR11280246	ERS14831650	ERA23129176	ERP001505	*Streptococcus pneumoniae*
ERR11280238	ERS14831639	ERA23129176	ERP001505	*Streptococcus pneumoniae*
ERR11280170	ERS14831807	ERA23129176	ERP001505	*Streptococcus pneumoniae*
ERR11280237	ERS14831687	ERA23129176	ERP001505	*Streptococcus pneumoniae*
ERR12768093	ERS17991890	ERA29530219	ERP001505	*Streptococcus pneumoniae*
ERR11280475	ERS14831634	ERA23129176	ERP001505	*Streptococcus pneumoniae*
ERR11280359	ERS14831726	ERA23129176	ERP001505	*Streptococcus pneumoniae*
ERR11280240	ERS14831643	ERA23129176	ERP001505	*Streptococcus pneumoniae*
ERR11280393	ERS14831736	ERA23129176	ERP001505	*Streptococcus pneumoniae*
ERR11280128	ERS14831794	ERA23129176	ERP001505	*Streptococcus pneumoniae*
ERR12768049	ERS17991908	ERA29530219	ERP001505	*Streptococcus pneumoniae*
ERR12908418	ERS17742562	ERA29605220	ERP001505	*Streptococcus pneumoniae*
ERR14365716	ERS22549746	ERA31142933	ERP001505	*Streptococcus pneumoniae*
ERR12768055	ERS17991915	ERA29530219	ERP001505	*Streptococcus pneumoniae*
ERR11010231	ERS14651719	ERA20775729	ERP001505	*Streptococcus_pneumoniae*
ERR13358796	ERS19862771	ERA30698859	ERP001505	*Streptococcus pneumoniae*
ERR11280270	ERS14831664	ERA23129176	ERP001505	*Streptococcus pneumoniae*
ERR11280338	ERS14831732	ERA23129176	ERP001505	*Streptococcus pneumoniae*
ERR12768103	ERS17991907	ERA29530219	ERP001505	*Streptococcus pneumoniae*
ERR11280344	ERS14831745	ERA23129176	ERP001505	*Streptococcus pneumoniae*
ERR11280220	ERS14831630	ERA23129176	ERP001505	*Streptococcus pneumoniae*
ERR12768102	ERS17991902	ERA29530219	ERP001505	*Streptococcus pneumoniae*
ERR12768075	ERS17991866	ERA29530219	ERP001505	*Streptococcus pneumoniae*
ERR11010249	ERS14651723	ERA20775729	ERP001505	*Streptococcus_pneumoniae*
ERR11582699	ERS15530218	ERA24269131	ERP001505	*Streptococcus pneumoniae*
ERR12768089	ERS17991884	ERA29530219	ERP001505	*Streptococcus pneumoniae*
ERR12908437	ERS17742566	ERA29605220	ERP001505	*Streptococcus pneumoniae*
ERR11280272	ERS14831668	ERA23129176	ERP001505	*Streptococcus pneumoniae*
ERR11280223	ERS14831678	ERA23129176	ERP001505	*Streptococcus pneumoniae*
ERR11280458	ERS14831632	ERA23129176	ERP001505	*Streptococcus pneumoniae*
ERR14365906	ERS22549787	ERA31142933	ERP001505	*Streptococcus pneumoniae*
ERR11280134	ERS14831796	ERA23129176	ERP001505	*Streptococcus pneumoniae*
ERR12908627	ERS17742568	ERA29605220	ERP001505	*Streptococcus pneumoniae*

ERR12768087	ERS17991883	ERA29530219	ERP001505	*Streptococcus pneumoniae*
ERR11010199	ERS14651701	ERA20775729	ERP001505	*Streptococcus_pneumoniae*
ERR12768107	ERS17991921	ERA29530219	ERP001505	*Streptococcus pneumoniae*
ERR14365844	ERS22549839	ERA31142933	ERP001505	*Streptococcus pneumoniae*
ERR11010227	ERS14651709	ERA20775729	ERP001505	*Streptococcus_pneumoniae*
ERR11280200	ERS14831816	ERA23129176	ERP001505	*Streptococcus pneumoniae*
ERR11280348	ERS14831749	ERA23129176	ERP001505	*Streptococcus pneumoniae*
ERR11280202	ERS14831815	ERA23129176	ERP001505	*Streptococcus pneumoniae*
ERR11010253	ERS14651724	ERA20775729	ERP001505	*Streptococcus_pneumoniae*
ERR11280302	ERS14831701	ERA23129176	ERP001505	*Streptococcus pneumoniae*
ERR12768100	ERS17991900	ERA29530219	ERP001505	*Streptococcus pneumoniae*
ERR11280485	ERS14831637	ERA23129176	ERP001505	*Streptococcus pneumoniae*
ERR12908417	ERS17742561	ERA29605220	ERP001505	*Streptococcus pneumoniae*
ERR11280340	ERS14831738	ERA23129176	ERP001505	*Streptococcus pneumoniae*
ERR12768041	ERS17991881	ERA29530219	ERP001505	*Streptococcus pneumoniae*
ERR11280333	ERS14831718	ERA23129176	ERP001505	*Streptococcus pneumoniae*
ERR14365626	ERS22549838	ERA31142933	ERP001505	*Streptococcus pneumoniae*
ERR11009892	ERS14310440	ERA20775729	ERP001505	*Streptococcus pneumoniae*
ERR12768084	ERS17991878	ERA29530219	ERP001505	*Streptococcus pneumoniae*
ERR11280425	ERS14831750	ERA23129176	ERP001505	*Streptococcus pneumoniae*
ERR11280427	ERS14831753	ERA23129176	ERP001505	*Streptococcus pneumoniae*
ERR12768045	ERS17991905	ERA29530219	ERP001505	*Streptococcus pneumoniae*
ERR12768088	ERS17991882	ERA29530219	ERP001505	*Streptococcus pneumoniae*
ERR11280351	ERS14831719	ERA23129176	ERP001505	*Streptococcus pneumoniae*
ERR11280449	ERS14831756	ERA23129176	ERP001505	*Streptococcus pneumoniae*
ERR11010313	ERS14651707	ERA20775729	ERP001505	*Streptococcus_pneumoniae*
ERR11010197	ERS14651699	ERA20775729	ERP001505	*Streptococcus_pneumoniae*
ERR11280387	ERS14831731	ERA23129176	ERP001505	*Streptococcus pneumoniae*
ERR11280301	ERS14831706	ERA23129176	ERP001505	*Streptococcus pneumoniae*
ERR12768048	ERS17991906	ERA29530219	ERP001505	*Streptococcus pneumoniae*
ERR11280160	ERS14831802	ERA23129176	ERP001505	*Streptococcus pneumoniae*
ERR11010229	ERS14651708	ERA20775729	ERP001505	*Streptococcus_pneumoniae*
ERR11280226	ERS14831821	ERA23129176	ERP001505	*Streptococcus pneumoniae*
ERR14365744	ERS22549754	ERA31142933	ERP001505	*Streptococcus pneumoniae*
ERR11280224	ERS14831817	ERA23129176	ERP001505	*Streptococcus pneumoniae*
ERR12768090	ERS17991885	ERA29530219	ERP001505	*Streptococcus pneumoniae*
ERR11280365	ERS14831729	ERA23129176	ERP001505	*Streptococcus pneumoniae*
ERR11280261	ERS14831695	ERA23129176	ERP001505	*Streptococcus pneumoniae*
ERR11280368	ERS14831752	ERA23129176	ERP001505	*Streptococcus pneumoniae*
